# The significance of CD16+ monocytes in the occurrence and development of chronic thromboembolic pulmonary hypertension: insights from single-cell RNA sequencing

**DOI:** 10.3389/fimmu.2024.1446710

**Published:** 2024-08-13

**Authors:** Maohe Chen, Qiuxia Wu, Nan Shao, Xingyue Lai, Huo Lin, Min Chen, Yijing Wu, Jiafan Chen, Qinghuang Lin, Jiahui Huang, Xiaoyun Chen, Wei Yan, Shi Chen, Hongli Li, Dawen Wu, Minxia Yang, Chaosheng Deng

**Affiliations:** ^1^ Department of Respiratory and Critical Care Medicine, First Affiliated Hospital of Fujian Medical University, Fuzhou, China; ^2^ Institute of Respiratory Disease, Fujian Medical University, Fuzhou, China; ^3^ Division of Critical Care Medicine, First Affiliated Hospital of Fujian Medical University, Fuzhou, China; ^4^ Department of Pulmonary and Critical Care Medicine, Shishi County Hospital, Shishi, China; ^5^ Department of Respiratory and Critical Care Medicine, Fuqing City Hospital Affiliated to Fujian Medical University, Fuzhou, China; ^6^ Department of Respiratory and Critical Care, Wuhan No. 6 Hospital, Affiliated Hospital of Jianghan University, Wuhan, China; ^7^ Department of Respiratory and Critical Care Medicine, National Regional Medical Center, Binhai Campus of the First Affiliated Hospital, Fujian Medical University, Fuzhou, China

**Keywords:** chronic thromboembolic pulmonary hypertension, chronic thromboembolic pulmonary disease, single cell RNA sequencing, CD16+ monocytes, macrophages, pulmonary hypertension

## Abstract

**Background:**

Chronic thromboembolic pulmonary hypertension (CTEPH) is a serious pulmonary vascular disease characterized by residual thrombi in the pulmonary arteries and distal pulmonary microvascular remodeling. The pathogenesis of CTEPH remains unclear, but many factors such as inflammation, immunity, coagulation and angiogenesis may be involved. Monocytes are important immune cells that can differentiate into macrophages and dendritic cells and play an important role in thrombus formation. However, the distribution, gene expression profile and differentiation trajectory of monocyte subsets in CTEPH patients have not been systematically studied. This study aims to reveal the characteristics and functions of monocytes in CTEPH patients using single-cell sequencing technology, and to provide new insights for the diagnosis and treatment of CTEPH.

**Methods:**

Single-cell RNA sequencing (scRNA-seq) were performed to analyze the transcriptomic features of peripheral blood mononuclear cells (PBMCs) from healthy controls, CTEPH patients and the tissues from CTEPH patients after the pulmonary endarterectomy (PEA). We established a CTEPH rat model with chronic pulmonary embolism caused by repeated injection of autologous thrombi through a central venous catheter, and used flow cytometry to detect the proportion changes of monocyte subsets in CTEPH patients and CTEPH rat model. We also observed the infiltration degree of macrophage subsets in thrombus tissue and their differentiation relationship with peripheral blood monocyte subsets by immunofluorescence staining.

**Results:**

The results showed that the monocyte subsets in peripheral blood of CTEPH patients changed significantly, especially the proportion of CD16+ monocyte subset increased. This monocyte subset had unique functional features at the transcriptomic level, involving processes such as cell adhesion, T cell activation, coagulation response and platelet activation, which may play an important role in pulmonary artery thrombus formation and pulmonary artery intimal remodeling. In addition, we also found that the macrophage subsets in pulmonary endarterectomy tissue of CTEPH patients showed pro-inflammatory and lipid metabolism reprogramming features, which may be related to the persistence and insolubility of pulmonary artery thrombi and the development of pulmonary hypertension. Finally, we also observed that CD16+ monocyte subset in peripheral blood of CTEPH patients may be recruited to pulmonary artery intimal tissue and differentiate into macrophage subset with high expression of IL-1β, participating in disease progression.

**Conclusion:**

CD16+ monocytes subset had significant gene expression changes in CTEPH patients, related to platelet activation, coagulation response and inflammatory response. And we also found that these cells could migrate to the thrombus and differentiate into macrophages with high expression of IL-1β involved in CTEPH disease progression. We believe that CD16+ monocytes are important participants in CTEPH and potential therapeutic targets.

## 1 Introduction

Chronic thromboembolic pulmonary hypertension (CTEPH) is a malignant pulmonary vascular disease. After acute pulmonary embolism, various factors such as inflammation and immunity may cause the thrombus not dissolving completely, and the undissolved thrombus undergoes fibrosis, causing narrowing and occlusion of the pulmonary artery lumen, which leads to progressive increase in pulmonary artery resistance and pressure, eventually leading to right ventricular dysfunction and death (1). More and more cohort studies on the chronicization of pulmonary embolism have found that some patients have symptoms of exertional dyspnea, and imaging shows obstructive lesions similar to CTEPH, but without resting pulmonary hypertension. Therefore, the European Respiratory Society has unified the name of this disease, namely chronic thromboembolic pulmonary disease (CTEPD), which may or may not be accompanied by pulmonary hypertension (2, 3).

The most effective treatment for CTEPH is pulmonary endarterectomy (PEA), but a part of patients cannot undergo this surgery, and 25% of patients still have residual pulmonary hypertension after surgery (3). However, the pathogenesis of CTEPH is still unclear. It is currently believed that there are two types of lesions in CTEPH, namely incomplete dissolution of thrombus causing proximal pulmonary vascular obstruction and secondary distal pulmonary microvascular remodeling (2). An increasing amount of research evidence indicates that the immune system and chronic inflammation play crucial roles in the pathogenesis of CTEPH (4–7). Histopathological findings in CTEPH patients also confirm extensive infiltration and accumulation of immune cells (8). Inflammation is one of the key mechanisms in thrombus formation, with monocytes, as primary immune cells, capable of early infiltration into thrombi. However, current research on monocytes primarily focuses on diseases such as deep vein thrombosis, stroke, and acute myocardial infarction (9–12), while studies on circulating monocytes in patients with CTEPH are scarcely reported.

Recent advancements in high-throughput sequencing technology, particularly single-cell sequencing, have enabled researchers to examine the transcriptome differences and differentiation trajectories among various cell subgroups at the single-cell level, enhancing our understanding of disease pathogenesis and identifying potential therapeutic targets. This study employs scRNA-seq to analyze the gene expression profiles and subsets of monocytes in CTEPH patients, along with scRNA-seq datasets from PEA specimens of CTEPH patients. This approach has helped reveal the differentiation scenarios of monocytes infiltrating tissues, potentially unveiling the unique role of monocytes in the pathophysiology of CTEPH and offering new perspectives and strategies for treating and managing the disease.

## 2 Method

### 2.1 Participants and clinical characteristics

The donors of this study were recruited from the First Affiliated Hospital of Fujian Medical University between March and December 2022. This study was approved by the Ethics Committee of the First Affiliated Hospital of Fujian Medical University (MRCTA, ECFAH of FMU (2022)174) and was conducted following the principles of the Helsinki Declaration. The donors signed the relevant informed consent forms. We included CTEPD patients according to the 2022 ERS/ESC pulmonary hypertension guidelines and excluded patients who were under 18 years old or could not provide informed consent. (**Supplementary Table 1**) showed the demographic and clinical characteristics of 5 CTEPH patients who provided peripheral blood scRNA-seq, and their radiological data were shown in (**Supplementary Figure 1**). In addition, we selected 1 healthy scRNA-seq donor and 15 healthy flow cytometry validation donors during routine physical examinations, who had no recent history of fever, infection or immune-related diseases.

### 2.2 Isolation of PBMCs and preparation of single-cell suspensions

For each participant, 2mL of venous blood was collected into an EDTA anticoagulant tube and transported to the laboratory on ice for scRNA-seq analysis. The blood was processed within 2 hours after collection. The PBMCs were isolated by density gradient centrifugation using Ficoll-Paque Plus medium (GE Healthcare) and washed with Ca/Mg-free PBS. 2 mL GEXSCOPE^®^ red blood cell lysis buffer (RCLB, Singleron) was added at 25°C for 10 min to remove the red blood cells. The solution was then centrifuged at 500 × g for 5 min and suspended in PBS. The blood samples were centrifuged at 400 × g for 5 min at 4°C, and the supernatant was discarded. After removing red blood cells, PBMCs were isolated by centrifugation at 400 × g for 10 min at 4°C. The supernatant was discarded and the PBMCs were resuspended by phosphate buffered saline to obtain a single-cell suspension.

### 2.3 Single-cell RNA preparation and sequencing

Single-cell suspensions (2×10^5^ cells/mL) were prepared using PBS (HyClone), and then the single-cell suspension was loaded onto a microwell chip using the Singleron Matrix^®^ Single Cell Processing System. GEXSCOPE^®^ Single Cell RNA Library Kits (Singleron) (13) were used for single-cell capture and library construction according to the manufacturer’s specifications. Sequencing was performed on the Illumina HiSeq X and Illumina Novaseq 6000 platforms.

### 2.4 Other data sources

Due to the COVID-19 pandemic that occurred during this study, and considering that many studies have reported that COVID-19 infection can affect the phenotype and function of peripheral blood immune cells, we included one healthy volunteer recruited before the COVID-19 pandemic as well as two single-cell sequencing samples of PBMCs from healthy controls (GSM4787557; GSM4787550) from the GSE158055 dataset in GEO database. The pulmonary artery endarterectomy tissue dataset GSE224143 of CTEPH patients was downloaded from GEO database. After preliminary assessment of the data, we found that the cell numbers of GSM7016384 and GSM7016387 samples were significantly lower than those of other samples (**Supplementary Figure 2**), so we only selected three samples with more than 2000 cells for joint data analysis.

### 2.5 Single-cell RNA sequencing data analysis

Single-cell expression data, including demultiplexing, genome alignment (GRCh38), barcode counting, and unique molecular identifier (UMI) counting, were processed using the CeleScope pipeline (v1.9.0, Singleron). FastQC (v0.11.9) (14) and Fastp (v0.23.2) (15) were used to remove low-quality reads. Cutadapt (v3.8) (16) was used to trim poly-A tail and adapter sequences. Then we used STAR (v2.6.1a) (17) to map reads to the reference genome GRCh38 (Ensembl version 92 annotation). FeatureCounts (v2.0.1) (18) was used to obtain the UMI count and gene expression level for each cell, and generate an expression matrix file for subsequent analysis.

### 2.6 Quality control, dimension-reduction and clustering (Scanpy)

Scanpy (19) (v1.8.1) was used for quality control, dimensionality reduction and clustering under Python (v3.7). For each sample dataset, we filtered expression matrix by the following criteria: 1) cells with gene count less than 200 or with top 2% gene count were excluded; 2) cells with top 2% UMI count were excluded; 3) cells with mitochondrial content > 10% were excluded; 4) genes expressed in less than 5 cells were excluded. After filtering, 77,501 cells were retained for the downstream analyses, with on average 1,557 genes and 3,904 UMIs per cell. The raw count matrix was normalized by total counts per cell and logarithmically transformed into normalized data matrix. Top 2000 variable genes were selected by setting flavor = ‘seurat’. Principle Component Analysis (PCA) was performed on the scaled variable gene matrix, and top 20 principle components were used for clustering and dimensional reduction. Cells were separated into 31 clusters by using Louvain algorithm and setting resolution parameter at 1.2. Cell clusters were visualized by using Uniform Manifold Approximation and Projection (UMAP).

### 2.7 Batch effect removal

Batch effect between samples was removed by Harmony (v1.0) using the top 20 principal components from PCA.

### 2.8 Differentially expressed genes analysis (scanpy)

To identify differentially expressed genes (DEGs), we used the scanpy.tl.rank_genes_groups function based on Wilcoxon rank sum test with default parameters, and selected the genes expressed in more than 10% of the cells in either of the compared groups of cells and with an average log2 (Fold Change) value greater than 0.25 as DEGs. Adjusted P value was calculated by Benjamini-Hochberg correction and the value 0.05 was used as the criterion to evaluate the statistical significance.

### 2.9 Pathway enrichment analysis

To investigate the potential functions of CD16+ monocytes, Gene Ontology (GO) and Kyoto Encyclopedia of Genes and Genomes (KEGG) analysis were performed with the “clusterProfiler” R package (v3.16.1) (20). Pathways with p_adj value less than 0.05 were considered as significantly enriched. Selected significant pathways were plotted as bar plots. GSEA (21) was performed to further elucidate the biological pathways enriched in specific cell types or states, providing additional insights into the observed functional differences. For GSVA pathway enrichment analysis, the average gene expression of each cell type was used as input data (22).

### 2.10 Cell-type recognition with Cell-ID

Cell-ID is a multivariate approach that extracts gene signatures for each individual cell and perform cell identity recognition using hypergeometric tests (HGT). Dimensionality reduction was performed on normalized gene expression matrix through multiple correspondence analysis, where both cells and genes were projected in the same low dimensional space. Then a gene ranking was calculated for each cell to obtain most featured gene sets of that cell. HGT were performed on these gene sets against brain reference from SynEcoSys database, which contains all cell-type’s featured genes. Identity of each cell was determined as the cell-type has the minimal HGT P value. For cluster annotation, Frequency of each cell-type was calculated in each cluster, and cell-type with highest frequency was chosen as cluster’s identity.

The cell type identification of each cluster was determined according to the expression of canonical markers from the reference database SynEcoSys™ (Singleron Biotechnology). SynEcoSys™ contains collections of canonical cell type markers for single-cell seq data, from CellMakerDB, PanglaoDB and recently published literatures.

### 2.11 SCENIC analysis

Pyscenic (v0.11.0) (23) was used to construct the transcription factor network. The GRNBoost2/GENIE3 was used to predict a regulatory network based on the co-expression of regulators and targets. CisTarget was then applied to exclude indirect targets and search for transcription factor binding motifs. Following that, AUCell was used for regulon activity quantification for every cell. Then the top TF regulons with high RSS (Regulon Specificity Score) were visualized using pheatmap in R (v4.3.1).

### 2.12 Pseudo-time trajectory analysis

Cell differentiation trajectory was reconstructed with Monocle2 (v2.22.0) (24). Highly-variable genes (HVGs) were used to sort cells in order of spatial‐temporal differentiation. We used DDRTree to perform FindVairableFeatures and dimension-reduction. Finally, The trajectory was visualized by plot_cell_trajectory function.

### 2.13 Ro/e analysis

To quantify the enrichment of cell clusters across groups, we compared the observed and expected cell numbers in each cluster by computing the Ro/e value using the χ2 test. We assumed that one cluster was enriched in a specific group if Ro/e > 1.

### 2.14 Animal models

All animal experiments were approved by the Animal Ethics Committee of Fujian Medical University, animal ethics number: IACUC FJMU2022-0442. The experiments were conducted in accordance with the Guide for the Care and Use of Laboratory Animals published by the US National Institutes of Health and the National Standard of the People’s Republic of China GB/T35892 - 2018 Guidelines for Animal Welfare and Ethical Review.

This experiment rats were SPF level, adult male, 6-8 weeks old, SD rats, weighing 300-350g (CTEPH model), weighing 200-250g (deep vein thrombosis model). All experimental animals were purchased from Shanghai Slik Experimental Animal Co., Ltd., which has an experimental animal license number: SCXK (Shanghai) 2022-0004. The purchased rats were kept in a 12-hour light-dark cycle, with the breeding room temperature maintained at 23 ± 3°C, and humidity maintained at about 60%, with free access for food and water.

Central venous catheterization CTEPH rat model construction: The rats were randomly divided into 2 groups (Control VS CTEPH).The rats were anesthetized with 3% sodium pentobarbital, and the left side of each rat’s neck was shaved and disinfected. A longitudinal incision about 1 cm long was made along the left side of the rat’s trachea at the clavicle midline, and the subcutaneous tissue was separated layer by layer. The left external jugular vein was isolated. A longitudinal incision about 1 cm long was made in the scapular region of the back, and a tunnel was made from the neck subcutaneously to the back subcutaneously with a hemostatic forceps. The central venous catheter was inserted through the back incision and brought out through the neck incision. The distal end of the freed left external jugular vein was ligated, and the proximal end was clamped with a vascular clamp and left with a suture for later use. The central venous catheter was inserted into the left external jugular vein. The catheter was fixed and the neck incisions were sutured. For the CTEPH group, autologous thrombi were prepared by blood sampling through the inserted catheter, and the autologous thrombi were injected through the central venous catheter daily for 3 days after catheterization. Tranexamic acid (200 mg/kg/d) was injected intraperitoneally throughout the process. At the same time, penicillin (100,000 U/kg/d) was injected intramuscularly to prevent infection after surgery. The control group used saline instead of autologous thrombus. Blood samples were collected through the central catheter on the 14th day after inserting catheter with three embolizations to detect the monocyte subsets of the peripheral blood by flow cytometry.

DVT rat model construction: The rats were randomly divided into 2 groups (Sham VS DVT). The rats were anesthetized with 3% sodium pentobarbital, shaved and disinfected their abdomen, spread towels, cut a longitudinal incision about 3 cm long at 1cm below the xiphoid process along the abdominal midline, cut open the abdominal muscles, exposed the abdominal cavity, separated the inferior vena cava below the left renal vein and ligated it, sutured the abdominal wall and skin layer by layer. Blood samples were collected through the tail vein on the 14th day after ligation to detect the changes of the peripheral blood monocyte subsets by flow cytometry.

### 2.15 Hemodynamic measurement, tissue collection and morphometric analysis

The rats were anesthetized and exposed the right necks, a PE-50 polyvinyl chloride catheter to a PowerLab (ADInstruments, Australia) multichannel physiological recorder. We pre-filled the catheter with heparin and inserted it into the right external jugular vein of the rats. The waveform data was recorded.

The excised left lung tissue (CTEPH model) and inferior vena cava (DVT model) were fixed in 4% polyformaldehyde, embedded in paraffin, and then cut into 4um sections. The sections were stained with HE and Masson. The sections were scanned with a Motic EASYSCAN instrument, observed and collected images with Motic DSAssistant software.

### 2.16 Immunofluorescence staining

After dewaxing the paraffin sections to water, antigen retrieval was performed with sodium citrate buffer. The sections were blocked with 5% BSA for 1 hour at room temperature. The following procedure was repeated for immunofluorescence multiplex staining: incubation with primary antibodies overnight at 4°C; incubation with corresponding secondary antibodies for 2 hours at room temperature; incubation with TSA staining working solution for 10 minutes. The nuclei were stained with DAPI staining solution and the slides were mounted with anti-fade mounting medium. Fluorescent images were acquired using a fluorescence microscope. The antibodies used were: CD68 (servicebio, GB113109, 1:3000), IL-1β (servicebio, GB11113, 1:3000), HRP conjugated Goat Anti-Rabbit IgG (H+L) (servicebio, GB23303, 1:500), iF488-Tyramide (servicebio, G1231, 1:500), Cy3-Tyramide (servicebio, G1223, 1:500).

### 2.17 Flow cytometry

The fresh peripheral blood was collected from CTEPD patients and healthy individuals, then density gradient centrifugation (Beijing Solarbio Science & Technology Co, Ltd, P8680) were performed at room temperature at 800 x g for 25 minutes, and the cells were collected at the white mononuclear cell interface. Then the cells were resuspend in the flow cytometer wash buffer (2% FBS in PBS) and stained with the following antibodies according to the standard protocol: CD14-PE (Elabscience, E-AB-F1209D) and CD16-FITC (Elabscience, E-AB-F1236C) for monocyte labeling. The gating strategy for quantifying the frequency of subcellular populations was as follows: in CD14+ cells, further divided monocyte subsets based on CD16 expression. We defined CD14+ monocytes as CD14+CD16-, CD16+ monocytes as CD14+CD16+, data were collected on a Biosciences AccuriC6 flow cytometer (BD Biosciences, USA), and analyzed using Flow Jo software (version 10.8).

The peripheral blood was collected from the rats, then density gradient centrifugation (Beijing Solarbio Science & Technology Co, Ltd, product number P6700) was performed at room temperature at 800 x g for 25 minutes, and the cells were collected from the white mononuclear cell interface. Then the cells were resuspend into the flow cytometer wash buffer (2% FBS in PBS) and stained with the following antibodies according to the standard protocol: CD172-BV421 (BD Biosciences, 744861), CD11b-APC (MULTI SCIENCES, F3101103) and CD43-FITC (BD Biosciences, 202814) for monocyte labeling. The gating strategy for quantifying the frequency of subcellular populations was as follows: in FSC-A, SSC-A gate, the monocyte population was identified, and FSC-A, FSC-H were used to exclude cell clumps. The total monocyte population was identified by high expression of CD11b+ and CD172+. Within the CD11b+ CD172+ cell population, the categorization of monocytes was based on the expression of CD43. Monocytes expressing CD43 were divided into two groups: those with CD43+ expression were labeled as CD172+CD11b+CD43+, while monocytes with higher CD43 expression (CD43++) were identified as CD172+CD11b+CD43++, data were collected on a Biosciences AccuriC6 flow cytometer (BD, USA), and analyzed using Flow Jo software (version 10.8).

### 2.18 Statistical analysis

Statistical analysis of the data was performed using R (version 4.3.1) and GraphPad Prism software (version 9.5). Categorical variables were expressed as counts and percentages (%), and continuous variables were expressed as mean ± SD. For comparison of two groups of data, Student’s t-test was used if the data followed a normal distribution; otherwise, Mann-Whitney U test was used. For comparison of categorical outcomes, chi-square test or Fisher’s exact test was used. All statistical tests were two-tailed, and p < 0.05 was considered statistically significant.

## 3 Result

### 3.1 ScRNA-seq reveals cellular landscape of peripheral blood and pulmonary artery endarterectomy tissue from CTEPH patients

**Figure 1:**
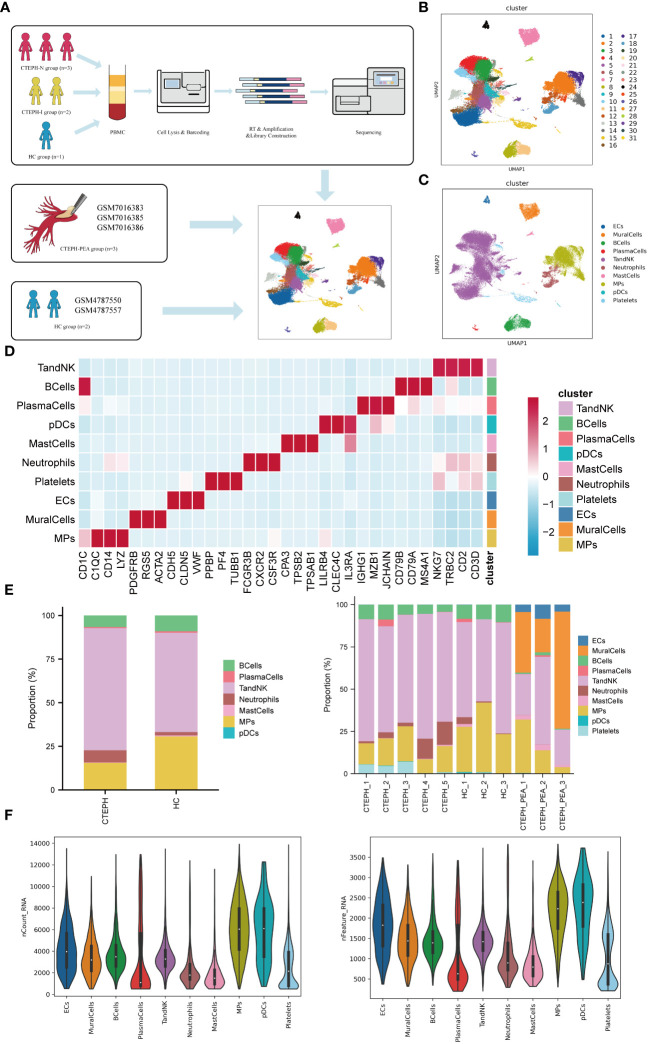
Single-cell RNA sequencing analysis of peripheral blood and pulmonary artery endarterectomy tissue from CTEPH patients. **(A)** The flowchart of the experimental process. (PBMC: peripheral blood mononuclear cell; HC group: healthy control group) **(B)** A UMAP plot of the clustering of 77,501 cells from peripheral blood and pulmonary artery endarterectomy tissue from CTEPH patients and healthy controls, revealing a total of 31 clusters. **(C)** UMAP plot showing each cell type in all samples. **(D)** Heatmap illustrating the prominent marker genes identified through single-cell RNA sequencing for the cell types defined in **(C)**. **(E)** A comparison of the percentages of different cell types in peripheral blood mononuclear cells (PBMCs) from CTEPH patients and healthy controls, as well as the proportion of cell types in each sample. **(F)** The transcript expression abundance of each cell type and the number of genes expressed in all cell types.

ScRNA-seq analysis was performed on PBMCs isolated from peripheral blood of 3 CTEPH patients who had not received any interventional treatment (CTEPH-N group), 2 CTEPH patients who had received interventional treatment (CTEPH-I group), and 3 healthy controls (HC group), as well as pulmonary artery endarterectomy tissues from 3 CTEPH patients (**Figure 1A**). After filtering out cells with high mitochondrial RNA, low RNA content, and doublets, a total of 77501 cells were obtained. We then performed unsupervised clustering of the cells, and identified 31 clusters and 10 cell types (**Figures 1B, C**) in all samples, including T/NK cells, Mononuclear Phagocyte system (MPs), B cells, plasma cells, endothelial cells, mural cells, mast cells, dendritic cells, neutrophils, and a small amount of platelets. The marker genes for each cell type, their projection on the UMAP plot, and the top 10 differentially expressed genes were shown in (**Figure 1D**, **Supplementary Figure 3**). The proportions of cell types between the CTEPH group and the healthy control group, as well as the composition of cell types within each sample, are illustrated in (**Figure 1E**). (**Figure 1F**) showed the transcript abundance and gene expression number of each cell type.

### 3.2 Proportion of peripheral blood monocyte subsets changed in CTEPH patients

**Figure 2:**
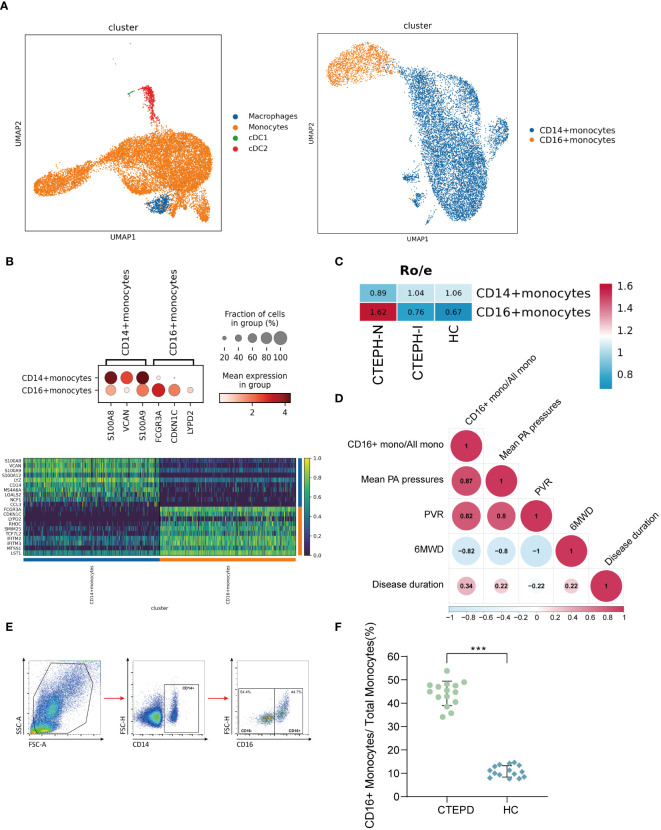
Changes in peripheral blood monocyte subsets in CTEPH patients. **(A)** UMAP plot of Mononuclear Phagocyte System (MPS) was divided into three cell types: monocytes, macrophages, and conventional dendritic cells (cDCs). Subsequently, monocytes were further divided into CD14+ monocytes and CD16+ monocytes. **(B)** A dot plot was used to annotate marker genes for CD14+ monocytes and CD16+ monocytes, along with a heatmap displaying the top 10 differentially expressed genes between the two cell types. **(C)** Ro/e (ratio of observed cell number to expected cell number) revealed the proportion of CD16+ monocytes in the CTEPH-N, CTEPH-I and HC groups. **(D)** The heat map showed the degree of correlation, and the numbers in the graph represented the correlation coefficient. (6MWD: 6-minute walk distance; PVR: pulmonary vascular resistance) **(E)** Schematic diagram of the gating strategy for distinguishing CD14+ monocytes from CD16+ monocytes by flow cytometry. **(F)** Differences in the proportion of CD16+ monocyte subsets in total monocytes between CTEPD patients (*n* = 15) and matched healthy controls (*n* = 15) were assessed by flow cytometry. All data were presented as means ± SEM, ^***^ *P* < 0.001.

We performed clustering analysis of the monocytes in the MPs of all samples, and divided them into two categories: CD14+ monocytes and CD16+ monocytes (**Figure 2A**). The marker genes and TOP10 differentially expressed genes of the two categories of monocyte subsets were shown in (**Figure 2B**), the marker genes, the projection of the marker genes on the UMAP plot, and the TOP10 differentially expressed genes of the four cell subpopulations in MPs were shown in (**Supplementary Figure 4**). We further analyzed the proportion of the two categories of monocyte subsets in the three groups of patients. Compared with the CTEPH-I group and the HC group, the Ro/e value of CD16+ monocytes in the peripheral blood of CTEPH-N patients was >1 (**Figure 2C**). The analysis results suggested that the proportion of CD16+ monocytes in the CTEPH-N group exceeded the normal expected level and may have biological significance. Interestingly, based on our data on the proportion of peripheral blood CD16+ monocytes in 5 CTEPH patients, we found that the proportion of CD16+ monocytes in CTEPH patients was positively correlated with mean pulmonary artery pressure (mPAP) (**Figure 2D**). MPAP is a critical measurement used to assess the severity of pulmonary hypertension. To verify the analysis results of single-cell data, we performed conventional flow cytometry to measure the proportion of CD14+ and CD16+ monocytes in 15 CTEPD patients and 15 paired healthy control groups. The standard flow cytometry gating strategy for annotating monocyte subsets was shown in (**Figure 2E**). The patient information included was shown in (**Supplementary Table 2**). The analysis results showed that the average percentage of CD16+ monocytes in CTEPD patients was higher than that in healthy control group (**Figure 2F**).

### 3.3 Functional characteristics of peripheral blood CD16+ monocytes in CTEPH patients

As mentioned above, our study results found that the proportion of CD16+ monocytes was significantly increased in CTEPH patients. This monocyte subset may play an important role in the pathogenesis of CTEPH. To explore the potential functional characteristics of CD16+ monocytes, we compared CD16+ monocytes with CD14+ monocytes from CTEPH patients (including CTEPH-N and CTEPH-I groups). Subsequently, we further compared CD16+ monocytes between the CTEPH group and the HC group.

**Figure 3:**
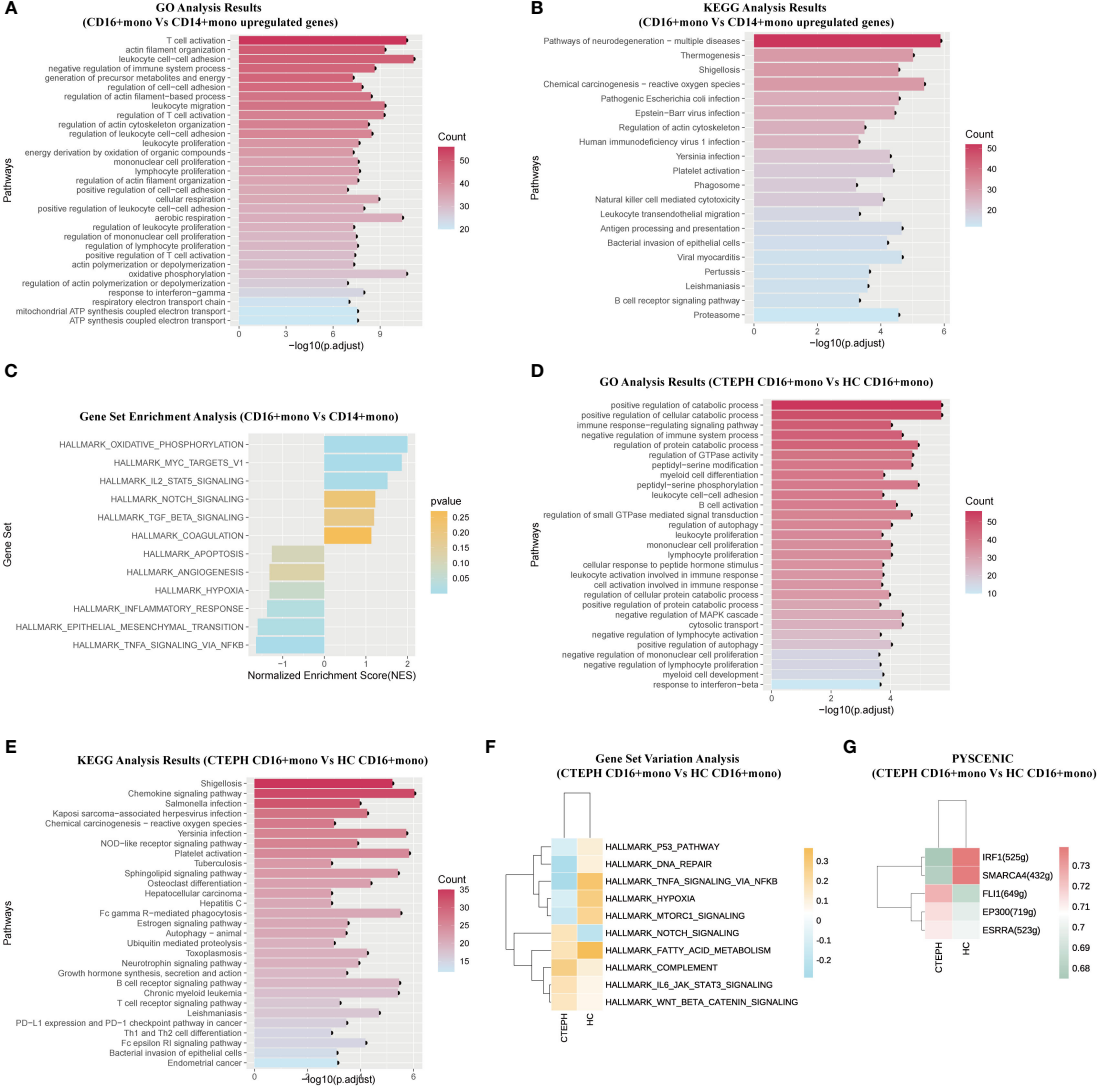
The functional characteristics of peripheral blood CD16+ monocytes in CTEPH patients. **(A)** GO analysis (biological process) of upregulated genes in CD16+ monocytes compared with CD14+ monocytes in CTEPH patients. **(B)** KEGG analysis of upregulated genes in CD16+ monocytes compared with CD14+ monocytes in CTEPH patients. **(C)** GSEA bar plot of CD16+ monocytes versus CD14+ monocytes in patients with CTEPH. **(D)** GO analysis (biological process) of upregulated genes in CD16+ monocytes between the CTEPH and healthy control samples. **(E)** KEGG analysis of upregulated genes in CD16+ monocytes between CTEPH patients and healthy controls. **(F)** GSVA heatmap of CD16+ monocytes between CTEPH patients and healthy controls. **(G)** Heat map of transcription factors upregulated in CD16+ monocytes between CTEPH patients and healthy controls.

Firstly, to understand the potential functions and roles of the two types of monocyte subsets in CTEPH patients, we performed GO and KEGG analysis of the transcriptome profiles of peripheral blood CD16+ monocytes and CD14+ monocytes from CTEPH patients to explore the possible biological functions and related signaling pathways of CD16+ monocytes in CTEPH disease. GO analysis results showed that “leukocyte cell-cell adhesion”, “T cell activation”, “positive regulation of T cell activation”, “positive regulation of cell-cell adhesion”, “regulation of leukocyte migration” and other terms were enriched in the CD16+ monocyte subset (**Figure 3A**). These results suggested that CD16+ monocytes may be involved in the interaction and immune regulation process between immune cells in CTEPH patients. Notably, most of the GO analysis results were enriched in T cell activation and cell adhesion. KEGG analysis results indicated that CD16+ monocytes from CTEPH patients might be related to transendothelial migration, and the analysis also showed that “Platelet activation” was closely related to CD16+ monocytes (**Figure 3B**). Gene set enrichment analysis (GSEA) of DEGs from the two monocyte subsets showed different characteristics, CD14+ monocytes were mainly enriched in “TNF-α signaling via NF-κB”, “inflammatory response” and “angiogenesis”, while CD16+ monocytes were mainly enriched in “IL2/STAT5 signaling”, “oxidative phosphorylation”, “TGF-β signaling” and “coagulation” (**Figure 3C**). The top 20 differentially expressed genes between CD14+ monocytes and CD16+ monocytes in CTEPH patients were shown in (**Supplementary Figure 5A**).

The above analysis results indicated that the two monocyte subsets had different biological functions, and compared with CD14+ monocytes, CD16+ monocytes had cell functions closely related to endothelial cell adhesion, T cell activation, coagulation response and platelet activation, and these biological functions had been proven to play a key role in thrombosis formation and chronic thromboembolic obstruction.

Secondly, considering the potential unique role of CD16+ monocytes in the pathophysiology of CTEPH, we compared the CD16+ monocytes from peripheral blood of CTEPH patients and healthy controls. The top 20 differentially expressed genes between CD16+ monocytes in CTEPH patients and the HC group were shown in (**Supplementary Figure 5B**). We found that the differentially expressed genes upregulated on CD16+ monocytes from CTEPH patients were enriched in pathways including “positive regulation of autophagy”, “leukocyte cell-cell adhesion” and “B cell activation” (**Figure 3D**). The KEGG analysis showed that signaling pathways and biological functions related to “Chemokine signaling pathway”, “Platelet activation” and “B cell receptor signaling pathway” were enriched in CD16+ monocytes of CTEPH patients (**Figure 3E**). GSVA showed that pathways related to COMPLEMENT, IL6-JAK-STAT3 signaling, NOTCH signaling and Wnt/β-catenin signaling were enriched in the CTEPH group, while pathways related to hypoxia and TNF-α signaling via NF-κB were significantly downregulated (**Figure 3F**). Previous studies have found that activated B cells were present in the thrombotic fibrotic and atherosclerotic lesions of the pulmonary artery intima in CTEPH, and there were few studies on the circulating and thrombus-resident B cells in CTEPH patients, but a well-known risk factor for CTEPH was splenectomy, considering the importance of the spleen for B cell maturation, pathogenic B cells may play a role in the pathogenesis of CTEPH (25). Through single-cell sequencing results, we observed that CD16+ monocytes of CTEPH patients might participate in the inflammatory response and thrombus formation of CTEPH by activating B cell receptor signaling-related pathways, B cell activation, platelet activation and their own chemotaxis migration.

Then, we applied pySCENIC analysis to identify transcription factors with differential gene expression between different CD16+ monocytes. Notably, genes regulated by FLI-1, EP300 and ESRRA were upregulated in CD16+ monocytes of CTEPH patients (**Figure 3G**).

### 3.4 Rat models of CTEPH and IVC successfully constructed both showed an increase in proportion of CD16+ monocytes

**Figure 4:**
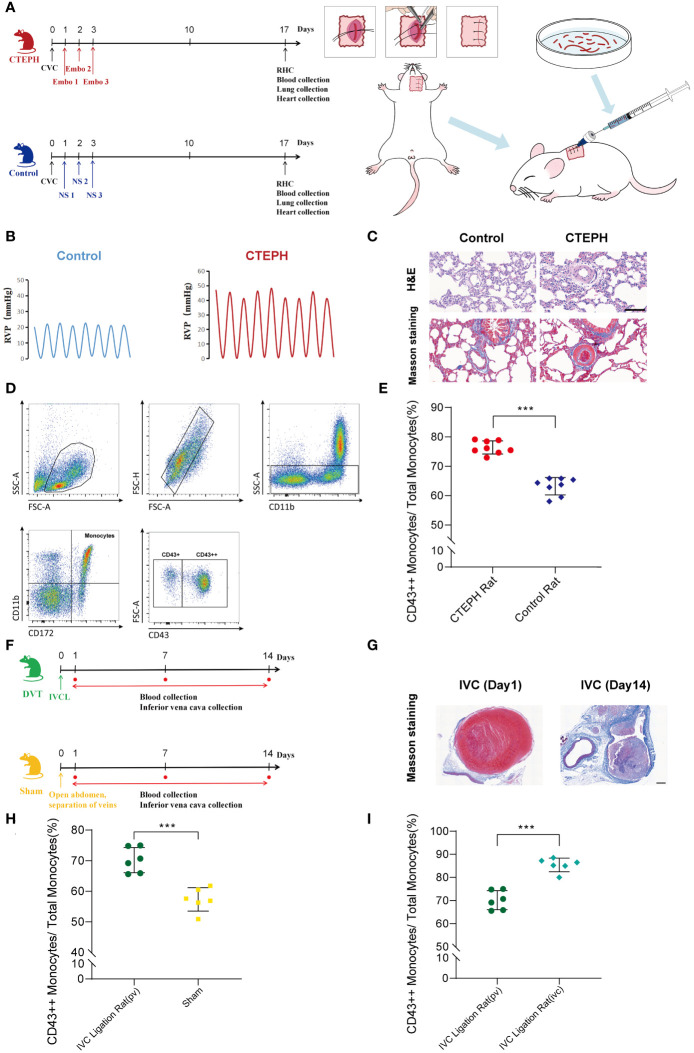
A change in the proportion of monocyte subsets similar to humans was found in animal models. **(A)** A flowchart of the CTEPH rat experiment design and a schematic diagram of the surgery for constructing the central venous catheter CTEPH rat model were shown. (CTEPH, Chronic Thromboembolic Pulmonary Hypertension; Ctrl, Control; CVC, Central Venous Catheterization; Embo, Embolism; NS, Normal Saline; RHC, Right Heart Catheterization). **(B)** Right heart catheterization to detect right ventricular pressure changes in rats in Control group and CTEPH group. (RVP: Right ventricular pressure). **(C)** Representative images of H&E and Masson staining of lung tissue from Control and CTEPH rats. Scale bar = 100 µm. **(D)** A schematic diagram of the flow cytometry gating strategy for distinguishing monocyte subsets in rat blood. **(E)** The difference in the proportion of CD43++ monocyte subsets in peripheral blood between the Control group rats (*n* = 8) and the CTEPH group rats (*n* = 8) was evaluated by flow cytometry. **(F)** Flowchart of IVC group rat experiment design. **(G)** Representative Masson-stained images of the inferior vena cava from a rat model of inferior vena cava ligation at day 1 and day 14 after modeling. Scale bar = 100 µm. **(H)** The difference in the proportion of CD43++ monocyte subsets in peripheral blood between the Sham group rats (*n* = 6) and the IVC group rats (*n* = 6) was evaluated by flow cytometry. **(I)** The difference in the proportion of CD43++ monocyte subsets in the peripheral blood (*n* = 6) and the inferior vena cava thrombus formation site (*n* = 6) of the IVC group rats was evaluated by flow cytometry. All data were presented as means ± SEM, ^***^ *P* < 0.001.

To further investigate the potential role and mechanism of CD16+ monocytes in the chronicization process of pulmonary embolism, we successfully constructed a CTEPH rat model which can be repeatedly embolized with the autologous thrombus (**Figure 4A**, **Supplementary Figure 6**). The indwelling central venous catheter could be repeatedly used for drawing blood and injecting autologous thrombus. This approach avoids the repeated puncture of the jugular vein, thereby reducing the risks of vascular damage and puncture failure. We dissected the model rats by finding that the surface of the lung tissue in the CTEPH group with multiple reddish-brown spots, which were ischemic manifestations caused by embolism and the heart was enlarged and pear-shaped, which was a cardiac change caused by increased pulmonary artery pressure leading to right ventricular enlargement (**Supplementary Figure 7**). We separated the pulmonary artery and found obvious thrombus residues in the blood vessels (**Supplementary Figure 8**). The right ventricular systolic pressure (RVSP) of CTEPH rats was significantly higher than that of the control group (**Figure 4B**, **Supplementary Figure 9**). To clarify whether pulmonary vascular remodeling occurred in CTEPH rats, we performed HE staining and Masson staining on the rat lung tissue. We found that in the CTEPH group, thrombus blocked the pulmonary artery, and the thrombus material was significantly thickened and fibrotic at the junction with the intima. Proximal blood vessels were blocked; distal microvessels had significant medial thickening, and distal pulmonary vessels were significantly muscularized. In addition, we found a large number of inflammatory cells infiltrating around the remodeled blood vessels, and the alveolar septum was significantly thickened (**Figure 4C**). This model suggested that it can simulate most of the pathophysiological manifestations of CTEPH patients.

Due to the species differences between rats and human beings, monocytes were heterogeneous in different species, and their surface molecular markers were also different (26). Human CD16+ monocyte subsets were functionally similar to rat CD43++ monocyte subsets (27, 28). Using this model, we analyzed rat peripheral blood by flow cytometry and observed that in CTEPH rats, CD43++ monocytes were significantly higher than those in sham-operated rats (**Figures 4D**, **E**).

Additionally, the inferior vena cava ligation rat model was considered to be one of the effective animal models for studying the chronic thrombosis fibrosis process of CTEPH. This model simulated the process of thrombus gradually undergoing dissolution, repair, and fibrosis in human CTEPH by ligating the inferior vena cava of rats. It could be used to study the mechanism of thrombus fibrosis, including fibrin synthesis, macrophage involvement, and fibroblast proliferation (1, 29–31). Therefore, we also constructed a deep vein thrombosis rat model with inferior vena cava ligation to verify our research findings (**Figure 4F**, **Supplementary Figure 10**). Masson staining of the inferior vena cava of rats 1 day after inferior vena cava ligation showed thrombus-like material inside, red, with a large number of red blood cells and platelets. Masson staining of the inferior vena cava of rats 14 days after inferior vena cava ligation showed fibrotic thrombus material blocking inside the blood vessel, mainly collagen-rich fibrotic lesions. A large number of neovascularization could be seen at the junction of thrombus and vein wall, indicating vascular recanalization lesions (**Figure 4G**). Using this model, we extracted blood from peripheral veins and from around thrombus formation below the inferior vena cava ligation point in rats, respectively. Flow cytometry analysis indicated that in the inferior vena cava ligation group rat model, the proportion of peripheral blood CD43++ monocytes was significantly higher than that in the sham operation group (**Figure 4H**). Interestingly, in the inferior vena cava ligation group rat model, the proportion of CD43++ monocytes in blood around thrombus formation in the inferior vena cava was also higher than that in peripheral blood (**Figure 4I**).

### 3.5 Most macrophages in the pulmonary endarterectomy tissue of CTEPH patients have pro-inflammatory characteristics and undergo metabolic reprogramming

Immune cells play an important role in the process of thrombus formation and dissolution, which has been confirmed (32, 33). Macrophages were the main immune cell type in the pulmonary endarterectomy tissue of CTEPH patients, and they were mainly distributed in areas containing thrombi (1, 6). To explore the impact of macrophage subsets and their functions on the changes of immune microenvironment in the pathogenesis of CTEPH, we performed single-cell RNA sequencing analysis of macrophages in three pulmonary endarterectomy tissues. Due to the small number of cells annotated to macrophages in one sample, we conducted scRNA-seq on only two CTEPH-PEA samples.

**Figure 5:**
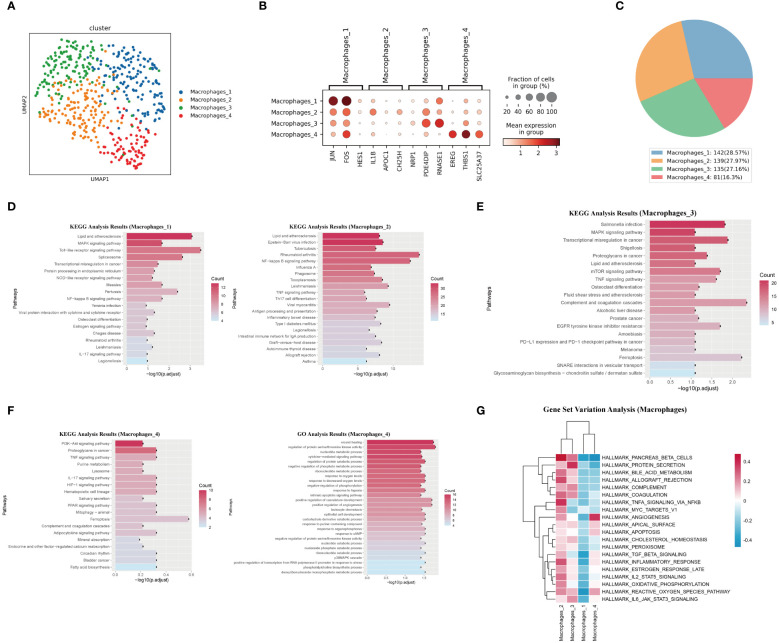
Characteristics analysis of macrophages in pulmonary artery endarterectomy specimens from CTEPH patients. **(A)** UMAP plot of four macrophage subpopulations. **(B)** TOP marker genes of four macrophage subpopulations. **(C)** Pie chart of proportions of 4 macrophage subpopulations. **(D, E)** KEGG analysis of differential genes in macrophages 1, macrophages 2 and Macrophages 3. **(F)** GO and KEGG analysis of macrophage 4 differential genes. **(G)** GSVA heatmap of 4 macrophage subpopulations.

We identified four macrophage subsets (**Figure 5A**). The marker genes for these macrophage subsets were shown in (**Figure 5B**), and their proportions in total macrophages were shown in (**Figure 5C**). The Macrophages 1 subset was characterized by the high expression of several genes, including JUN, FOS, and HES1. The Macrophages 2 subset was defined by the high expression of genes including IL-1β, APOC1, and CH25H. KEGG analysis results for these two subsets suggested their involvement in lipid metabolism and inflammatory response processes (**Figure 5D**). Macrophages 3 subset was characterized by the high expression of genes such as NRP1, PDE4DIP, and RNASE1. KEGG analysis of differentially expressed genes of this subset suggested that it was related to the complement and coagulation cascade and ferroptosis (**Figure 5E**). The Macrophages 4 subset showed high expression of genes like EREG, THBS1, and SLC25A37. This subset also had enriched genes related to the HIF-1 signaling pathway, PPAR signaling pathway, and ferroptosis. GO analysis results for this macrophage subset indicated enrichment in terms such as wound healing, positive regulation of angiogenesis, and positive regulation of vasculature development (**Figure 5F**). It was worth noting that, except for the Macrophages 4 subset, the remaining three subsets all enrichment in genes related to “Lipid and atherosclerosis” “lipid metabolism” and “atherosclerosis”.

GSVA analysis revealed the functional differences between the four macrophage subsets. The Macrophages 4 subset had the strongest angiogenic ability, while the Macrophages 2 and Macrophages 3 subsets exhibited obvious pro-inflammatory characteristics and were closely related to complement pathway and coagulation pathways (**Figure 5G**). The TOP10 differentially expressed genes, the projection of the macrophage subset marker genes on the UMAP plot, transcript abundance and gene expression number of each subset of macrophages were shown in (**Supplementary Figures 11**, **12**).

In summary, Macrophages 1, 2, and 3 subsets exhibited typical pro-inflammatory characteristics and were closely related to lipid metabolism and atherosclerosis. In contrast, the Macrophages 4 subset showed different characteristics from the other three subsets. Its activation of the PPAR signaling pathway and angiogenic characteristics reflect its anti-inflammatory nature. Therefore, in the chronic organized thrombus tissue of CTEPH patients, most macrophages exhibited pro-inflammatory characteristics and underwent lipid metabolic reprogramming.

### 3.6 Macrophages with high expression of IL-1β in the endarterectomy tissue of pulmonary artery may originate from peripheral blood CD16+ monocytes

The aforementioned analysis results indicated that the migration and adhesion abilities of peripheral blood CD16+ monocytes in CTEPH patients were significantly enhanced, suggesting that they might be recruited to the pulmonary arterial intima tissue and potentially participated in the development of the disease. At the same time, we observed that their autophagy function was significantly increased, which might be related to their differentiation into macrophages.

**Figure 6:**
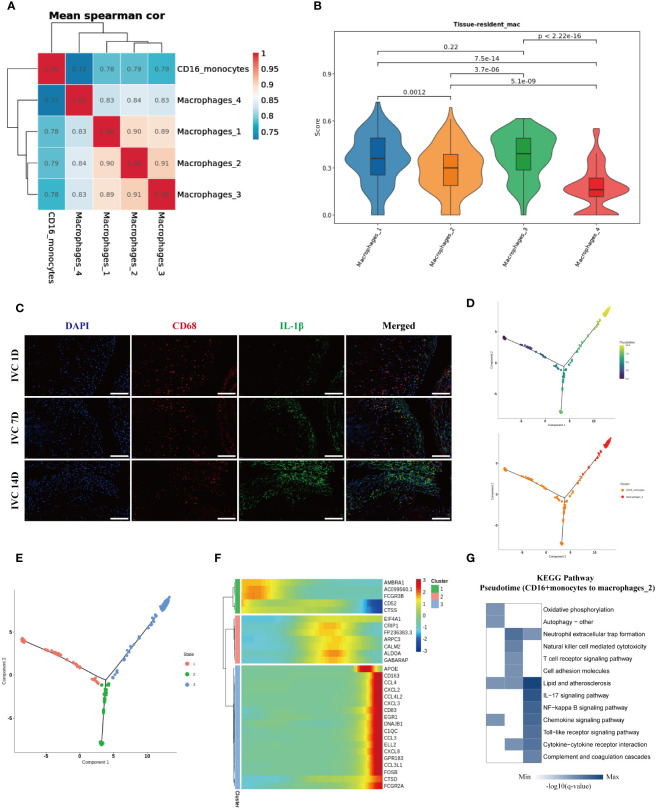
Characteristics analysis of CD16+ monocytes differentiation into IL-1β+ macrophages in peripheral blood of CTEPH patients. **(A)** Heatmap shows the correlation of CD16+ monocytes with four macrophage subgroups. **(B)** Resident macrophage scores of four macrophage subgroups. **(C)** Immunofluorescence images of CD68 (red), IL-1β (green) and DAPI (blue) in inferior vena cava thrombus tissue at different time points (1 day, 7 days and 14 days after ligation). Scale bar = 300 µm. **(D)** Pseudotime analysis of the developmental trajectory of CD16+ monocytes and Macrophages 2. **(E)** The developmental trajectory of CD16+ monocytes and Macrophages 2 was clustered into three clusters. **(F)** Genes with significant expression changes along the pseudotime trajectory. **(G)** KEGG pathway enrichment analysis along the pseudotime of CD16+ monocytes differentiation into Macrophages 2.

To explore the potential differentiation relationship between peripheral blood CD16+ monocytes and macrophage subgroups in the endarterectomy tissue of pulmonary arteries, we performed correlation analysis and found that peripheral blood CD16+ monocytes had the highest correlation with Macrophages 2 subgroup, which enriched genes such as IL-1β, APOC1, and CH25H, while the Macrophages 1 and Macrophages 3 subgroups, which enriched genes such as JUN and NRP1, also showed high correlation (**Figure 6A**). Furthermore, we used the tissue-resident macrophage-related gene set (34–38) (**Supplementary Table 3**) to evaluate the nature score of each macrophage subgroup and found that the Macrophages 1 and Macrophages 3 subgroups had higher resident macrophage scores, suggesting that they might be resident macrophage subgroups in the pulmonary arterial intima tissue. The Macrophages 2 and Macrophages 4 subgroups had lower resident macrophage scores, suggesting that they might be macrophage subgroups derived from peripheral blood monocytes (**Figure 6B**). Based on these analysis results, we inferred that the Macrophages 2 subgroup, which enriched IL-1β, APOC1, CH25H, and other genes, might be derived from peripheral blood CD16+ monocytes. We used the inferior vena cava ligation rat model to simulate the chronic fibrosis process of pulmonary arterial intima thrombus in CTEPH patients and used immunofluorescence staining to verify the infiltration of IL-1β+ macrophage subgroup in chronic thrombus. The immunofluorescence staining results suggested that over time, the number of infiltrated IL-1β+ macrophages in the thrombus gradually increased (**Figure 6C**). Therefore, these results suggested that IL-1β+ macrophage subgroup accumulates in chronic thrombus formation and might originate from CD16+ monocyte subgroup increased around thrombus. To further clarify the differentiation characteristics of peripheral blood CD16+ monocytes into IL-1β+ macrophages in CTEPH patients, we performed pseudotime analysis on CD16+ monocytes and IL-1β+ macrophages in CTEPH group and CTEPH-PEA group. The analysis results indicated that peripheral blood CD16+ monocytes in CTEPH patients were located at the starting point of the trajectory path, while Macrophages 2 with high expression of IL-1β in PEA tissue were located at the end of the trajectory path (**Figure 6D**). Additionally, we further analyzed the changes in key macrophage marker genes (CD68, CD163, MRC1) during the pseudotime analysis. The analysis results showed that the expression of these marker genes gradually increased over the developmental trajectory (**Supplementary Figure 13**). This supported the hypothesis that CD16+ monocytes differentiate into macrophages over time. We divided the cells on the differentiation trajectory into three clusters and found that with the progress of differentiation, genes related to lipid metabolism and chemokines were significantly upregulated in CD16+ monocytes (**Figures 6E**, **F**). We also ranked the pathway enrichment of cells according to pseudotime and found that CD16+ monocytes participated in different pathways at different stages of differentiation, including early “oxidative phosphorylation” and “autophagy”, middle “neutrophil extracellular trap formation”, “natural killer cell mediated cytotoxicity”, and late stages involving “lipid and atherosclerosis”, “chemokine signaling pathway”, and “complement and coagulation cascades” (**Figure 6G**). These results suggested that genes related to lipid metabolism and chemokines gradually increased in the process of CD16+ monocytes differentiation into IL-1β+ macrophages, and that CD16+ monocytes played different roles at different stages of differentiation.

## 4 Discussion

Miao et al. (39) analyzed the PEA tissue of 5 CTEPH patients by single-cell sequencing technology, and the results revealed the order of different types of cells appearing along the developmental trajectory in the development process of CTEPH. Notably, the results of this study suggest that immune cells may play an important role in the early development of CTEPH. Previous studies by Quarck et al. (6) suggested that there was a large amount of immune cell infiltration in PEA specimens of CTEPH patients, especially in thrombi and atherosclerotic lesions. Immune cells contribute to the disease process by releasing inflammatory cytokines, which not only promote inflammation but also activate the coagulation system and influence vascular remodeling, thereby driving the progression of CTEPH (40–42).

Monocytes, a type of immune cell that can differentiate into macrophages and dendritic cells (43), are generally divided into three different phenotypic and functional subsets: classic monocytes (CD14++CD16-) (CMs), intermediate monocytes (CD14++CD16+) (IMs), and non-classic monocytes (CD14+CD16++) (NCMs). IMs and NCMs are often collectively referred to as CD16+ monocytes, accounting for about 10%-20% of monocytes, with their proportion increasing to 20%-30% during periods of inflammation or immune response (44–46). In addition to their immune function, monocytes participate in thrombus formation by releasing tissue factor (TF) and forming circulating monocyte-platelet aggregates (MPA) with platelets (47, 48). According to a study by Allison et al. (49), monocytes can switch from an innate immune function phenotype to a pro-thrombotic phenotype in moderate COVID-19 patients, with monocytes upregulating the expression of pro-thrombotic genes after SARS-CoV-2 infection. Changes in the number of monocyte subsets in thrombotic diseases are related to the diagnosis and prognosis of these disease (50–52). Monocytes are an important factor that cannot be ignored in the development of CTEPH; however, specific research on them in the field of CTEPH is currently limited (31). In this study, we utilize single-cell RNA sequencing technology to analyze the roles of different monocyte subgroups in CTEPH. Given that intermediate and non-classical monocytes display similar functional behaviors in various inflammatory diseases, such as participating in inflammation responses, migrating to inflamed sites, and contributing to tissue remodeling (53–55), we have chosen to combine these two subgroups for analysis. This approach allows us to explore their potential synergistic and overlapping functions in CTEPH while mitigating the impact of insufficient sample sizes on the validity of statistical analysis. Although single-cell technology provides us with high-resolution data, considering the scarcity and biological proximity of these subgroups, a combined analysis is considered a reasonable strategy. This approach not only increases the statistical power of the analysis but also simplifies the model, allowing us to focus on the collective impact of these subgroups in the disease mechanism. Future research will further utilize single-cell technology to independently assess the specific contributions of each subgroup and more accurately elucidate their independent and combined roles in the pathogenesis of CTEPH.

Our single-cell sequencing studies revealed that the proportion of CD16+ monocyte subgroups in the peripheral blood of CTEPH patients was higher than that of the control group. Furthermore, we observed that after receiving interventional surgery, the proportion of CD16+ monocytes in CTEPH patients with improved pulmonary hemodynamics decreased significantly, which is consistent with Mylvaganam’s study (56). Their study results found that the average percentage of peripheral blood non-classical monocytes (CD14+CD16++) in CTEPH patients was higher than that in CTEPD patients and the control group, and the proportion of non-classical monocytes in CTEPH patients who did not undergo PEA surgery was higher than that in CTEPH patients who underwent PEA surgery. We further verified our research results using traditional flow cytometry and found that the proportion of CD16+ monocytes in CTEPD patients (some of whom were clearly diagnosed as CTEPH patients) was significantly higher than that in matched healthy controls. We observed the same phenomenon in our constructed CTEPH rat model and inferior vena cava ligation rat model, where CD16+ monocytes showed a significant upward trend. Notably, in the inferior vena cava ligation rat model, we found that the proportion of CD16+ monocytes in the blood at the site of thromboembolism was significantly higher than at the site where no thrombus was formed. This observation suggests that more CD16+ monocytes might be recruited to the thrombus, or that monocyte subgroups were recruited to the thrombus site and their phenotype changed under the influence of the thrombus microenvironment. According to the current guidelines for CTEPH (57), particularly the requirement for right heart catheterization—where after at least three months of anticoagulation therapy, the patient’s pulmonary artery pressure must be confirmed to exceed 20mmHg through a right heart catheterization—this invasive diagnostic method might lead many patients to refuse the procedure, thereby affecting the diagnosis rate of CTEPH. In light of this, our research proposes a new hypothesis: Could CD16+ monocytes be used as clinical biomarkers in conjunction with imaging results to diagnose CTEPH? This method could offer a less invasive and more acceptable diagnostic option for patients. Additionally, the potential uses of CD16+ monocytes might not be limited to diagnosis; they could also serve as predictors of treatment outcomes and patient prognosis. Future research across more centers and involving a broader patient cohort could further validate the role of CD16+ monocytes in clinical practice, potentially providing dual functions for the diagnosis and management of CTEPH. This approach not only promises to simplify the diagnostic process but also aims to enhance patient acceptance and compliance with recommended diagnostic evaluations, potentially improving the accuracy of CTEPH diagnosis and the timeliness of treatment optimization.

In earlier studies, some scholars have proposed that the pathological process of CTEPH is not only related to thrombus formation, but also related to thrombus dissolution disorder (58–60). In animal studies, it was found that by depleting CD11b+Ly6C^Low^ monocytes (similar to human CD16+ monocyte function) in chlorophosphate-treated inferior vena cava ligation mice, thrombus dissolution was significantly slowed down. After the injection of CD11b+Ly6C^Low^ monocytes, there was evident thrombus dissolution, leading to a significant reduction in thrombus volume. This suggests that CD11b+Ly6C^Low^ monocytes participate in and promote thrombus dissolution (61). However, our study found that CD16+ monocytes in CTEPH patients exhibited characteristics related to platelet activation and coagulation response. These results suggest that CD16+ monocytes in the peripheral blood of CTEPH patients are significantly involved in thrombus formation, indicating that the impaired function of CD16+ monocytes in CTEPH patients may contribute to a disorder in thrombus dissolution.

While it is known that macrophages exist in the organized thrombus and atherosclerotic lesions of CTEPH patients, a detailed characterization of macrophage subtypes in CTEPH patients remains lacking. Now, with the advances of high-throughput single-cell sequencing technology and bioinformatics analysis, we can depict the macrophage subtypes in the pulmonary artery thrombus tissue of CTEPH patients at single-cell resolution and study them. Miao et al. (39) depicted the immune cell types in the endarterectomy tissue of CTEPH patients by single-cell sequencing, and preliminarily analyzed the functions of macrophages, but they did not further subdivide the macrophage subtypes in their study. We analyzed the single-cell dataset of thrombus tissue from CTEPH patients provided by Viswanathan et al. In our analysis, we identified four macrophage subtypes. These subtypes are consistent with four of the seven macrophage subtypes described in the study published by Viswanathan et al. (42). The three macrophage subtypes that we did not identify may be due to our exclusion of samples with fewer cells from the dataset during the analysis process. Interestingly, Viswanathan et al. considered the macrophage subtype with high expression of genes such as JUN and FOS as an anti-inflammatory macrophage subtype in their study based on the results of Ingenuity Pathway Analysis. However, in our study, we performed enrichment analysis of DEGs for four subtypes of macrophages, and found that Macrophages 1 (with high expression of JUN, FOS, HES1 and other genes) were enriched in “Toll-like receptor signaling pathway” “NF-κB signaling pathway” “NOD-like receptor signaling pathway” and other inflammatory signaling pathways. Activation of the TLR signaling pathway prompts immune cells, including macrophages and dendritic cells, to produce inflammatory mediators like cytokines and chemokines, which guide the migration and activation of other immune cells, forming an inflammatory response. These findings suggest that Macrophages 1 is a pro-inflammatory macrophage subtype. Among the four macrophage subtypes that we identified in CTEPH thrombus tissue, the Macrophages 4 cluster exhibited significantly different characteristics compared to other macrophages. Gene Set Variation Analysis results indicated that it had the strongest angiogenic ability, and DEGs enrichment analysis of the Macrophages 4 subtype showed associations with wound healing and positive regulation of angiogenesis, suggesting that the Macrophages 4 subtype has anti-inflammatory and repair functions.

When encountering inflammatory signals, peripheral blood CD16+ monocytes can be activated to produce inflammatory responses and can also migrate to tissues and differentiate into macrophages (62). During thrombus formation, monocytes can be recruited to newly formed thrombi within blood vessels by chemokines (63). In our study, we observed that the proportion of peripheral blood CD16+ monocytes in CTEPH patients was increased, and the autophagy and migration functions of CD16+ monocytes were significantly upregulated. These findings suggest that peripheral blood CD16+ monocytes in CTEPH patients may be recruited to the thrombus site during thrombus formation and further differentiated into macrophages in thrombus tissue. By using gene correlation analysis and excluding resident macrophages, we found that IL-1β+ macrophages in thrombus tissue may originate from peripheral blood CD16+ monocytes and further validated our findings in animal experiments. When exploring the transcriptional features of CD16+ monocyte differentiation process by pseudotime analysis, we found that their lipid metabolism and chemokine-related gene expression gradually increased. Pseudotime pathway enrichment analysis revealed that mid-differentiation CD16+ monocytes were enriched in pathways related to neutrophil extracellular traps (NETs) formation, with this enrichment persisting into the late differentiation stage. The role of NETs in thrombotic diseases and CTEPH has been widely reported (64–67), and Sharma et al (31). Reported an interesting phenomenon that NETs can promote the transdifferentiation of circulating monocytes into fibroblasts in CTEPH patients. Our study results suggest that CD16+ monocyte subset may also play an important role in NETs formation. The interaction and influence mechanism between CD16+ monocytes and NETs warrant further exploration in future research.

Previous studies have reported the presence of macrophage-derived foam cells and cholesterol clefts in the atherosclerotic lesions in CTEPH patients (6, 68). Liu’s (69) study found that atherosclerotic lesion formation in CTEPH was not related to traditional risk factors for atherosclerosis, but CTEPH patients with atherosclerotic lesions usually had higher symptomatic embolism ratio and longer disease course. Pathological examination of endarterectomy tissue revealed typical lipid deposition below the thrombus, with areas of atherosclerosis also showing vascular generation defects. The researchers speculated that atherosclerosis formation was a secondary lesion caused by chronic fibrotic clots. In our study, among the four macrophage subtypes annotated in CTEPH thrombus tissue, three subtypes exhibited high expression of genes related to lipid metabolism and atherosclerosis, while only a few macrophages were pro-angiogenic. Macrophages are a group of highly plastic immune cells that are reprogrammed by metabolism, and previous studies have reported that lipid metabolism specifically influences their differentiation and function (70, 71). Our study supports Liu’s conjecture that macrophages in thrombus tissue underwent lipid metabolism reprogramming, leading to secondary atherosclerotic changes and impaired vascular generation. Decades of research have shown that foam cells in atherosclerosis originate from peripheral blood monocytes, and the secretion of the IL-1β cytokine by macrophage has been proven to be a main driving factor of atherosclerosis pathogenesis (72). Therefore, combined with our research results, in CTEPH atherosclerotic lesions, the IL-1β+ macrophage subtype derived from peripheral blood CD16+ monocytes play a major contributing role. This macrophage subtype not only highly expresses IL-1β, but also highly expresses APOC1. APOC1 is a small-volume lipoprotein that participates in cholesterol metabolism and lipid metabolism (73). Studies have shown that the presence of APOC1 can enhance the size and inflammatory state of atherosclerotic lesions in mice (74). APOC1 is also closely related to the infiltration of various immune cells in various cancers (75), indicating that this macrophage subtype also has potential immune regulatory functions.

This study has some limitations. We encountered the full-scale outbreak of COVID-19 in China during our research process. Considering the impact of COVID-19 infection on peripheral blood immune cells, especially monocyte subsets (76–80), in terms of quantity and function, we selected PBMC data from healthy control group published in databases for comparison analysis to ensure the accuracy and rigor of the study. However, since we did not have accurate baseline information data of the healthy control group, we could not rule out the influence of confounding factors such as age, gender and BMI on the study results. Since CTEPH is a relatively rare disease, and only a few centers in China and even worldwide can perform PEA surgery, we could only obtain single-cell sequencing results of PEA tissue from data provided by previous researchers. Although we used algorithms to remove batch effects, these measures may still have some impact on the study results. In this study, we choose to extract RNA from total PBMCs for gene expression analysis, rather than from specific monocyte subgroups (such as CD14+ and CD16+ monocytes) separated by flow cytometry. This decision is based on multiple considerations involving sample representativeness, technical limitations, and experimental feasibility. Analyzing total PBMCs preserves all intercellular interactions and signaling pathways, providing a comprehensive biomarker map that aids in observing the overall response of various cell types in the disease state. This information might be lost when analyzing isolated subgroups, especially when the interactions and signaling between cell types are crucial in immune responses, making the integrity of the sample particularly important. Although isolating each subgroup could provide more detailed data, this method is technically demanding and may affect the original biological state of the cells due to the sorting process. Given that CD16+ monocytes constitute a lower proportion of total monocytes, high-purity sorting poses a challenge and requires a larger initial sample volume to obtain enough cells for subsequent analysis. Additionally, flow cytometry sorting involves complex technical operations that could reduce cell viability, impacting the reliability of experimental results. Although this study represents the largest single-cell sequencing dataset analysis in the CTEPH research field to data, the high cost of single-cell sequencing technology means the sample size for scRNA-seq remains relatively small. In the future, larger-scale multicenter prospective studies and single-cell sequencing of CTEPH patients should be conducted to verify the role and mechanism of CD16+ monocytes in chronic pulmonary embolism.

The lack of suitable animal models has been a significant barrier to advancing research into the pathophysiological mechanisms of chronic thromboembolic pulmonary hypertension. Building on preliminary studies, our team has developed a more representative CTEPH rat model using central venous catheterization and repeated autologous thrombus injections. This model partially replicates the pathological changes caused by chronic thromboembolic events, such as pulmonary vascular remodeling. However, there are limitations; for instance, while thrombi obstructing the pulmonary arteries can be anatomically identified in the rats, the pathological changes do not fully replicate the chronic thromboembolic events seen in humans, such as significant thickening of the pulmonary artery intima. Additionally, due to the animals’ strong fibrinolytic abilities, most thrombi in the model rats dissolve completely or partially, posing significant challenges to studying the chronic fibrosis of thrombotic tissue. Therefore, we employed an inferior vena cava ligation rat model to simulate the chronic fibrotic process of thrombotic tissue and further our research with these two animal models. The primary focus of our study is CD16+ monocytes, which only constitute 10%-20% of the monocyte population. Despite repeated attempts at magnetic bead separation and flow cytometry to isolate CD16+ monocytes, we were unable to obtain a sufficient quantity of cells for subsequent *in vitro* experiments. Moreover, current technological methods make it challenging to specifically target, intervene, or deplete CD16+ monocytes within an animal’s body. This limitation hinders our ability to further explore the specific mechanisms by which CD16+ monocytes contribute to thrombosis formation through *in vitro* experiments and animal models, as well as to validate changes in signaling pathways identified by single-cell sequencing analysis.

In summary, we found the potential contribution of CD16+ monocytes to the pathogenesis of CTEPH. Compared with CD16+ monocytes from healthy individuals, they showed significant transcriptional changes, which may lead to platelet activation, enhanced coagulation response, and production of cytokines and chemokines in CTEPH patients. In addition, CD16+ monocytes had a migratory phenotype, indicating that they were recruited more to thrombus tissue and further differentiated into IL-1β+ macrophages and promoted atherosclerotic pathological changes. Therefore, intervening in the formation and differentiation process of CD16+ monocytes may be a target for improving the disease progression and treatment of CTEPH patients.

## Data Availability

The datasets presented in this study can be found in online repositories. The name of the repository and accession number can be found below: Gene Expression Omnibus; GSE274381.
