# Supplementary material for: The significance of CD16+ monocytes in the occurrence and development of chronic thromboembolic pulmonary hypertension: insights from single-cell RNA sequencing
Source: Front Immunol. 2024 Aug 13;15:1446710. doi: 10.3389/fimmu.2024.1446710 (PMC11347785; doi:10.3389/fimmu.2024.1446710)
Supplement: Supplementary file 1 [file Datasheet1.pdf]

Supplementary Figures

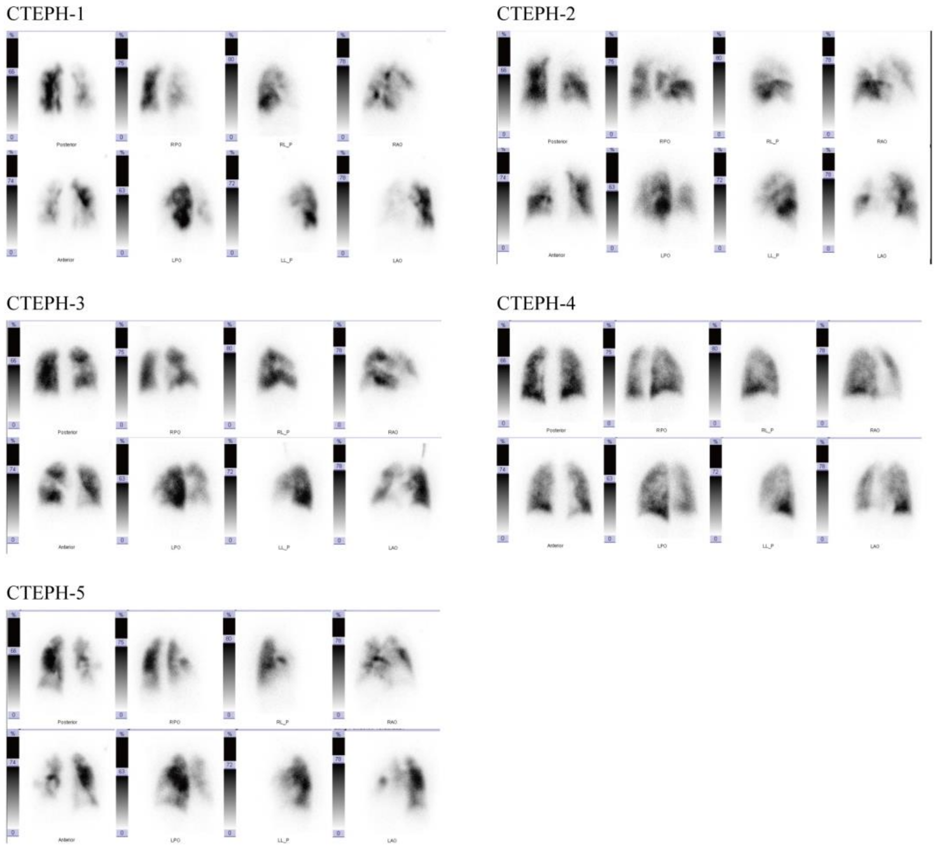

Supplementary Figure1. Ventilation-perfusion (VQ) scan results of 5 CTEPH patients who provided peripheral blood for scRNA-seq in this study.

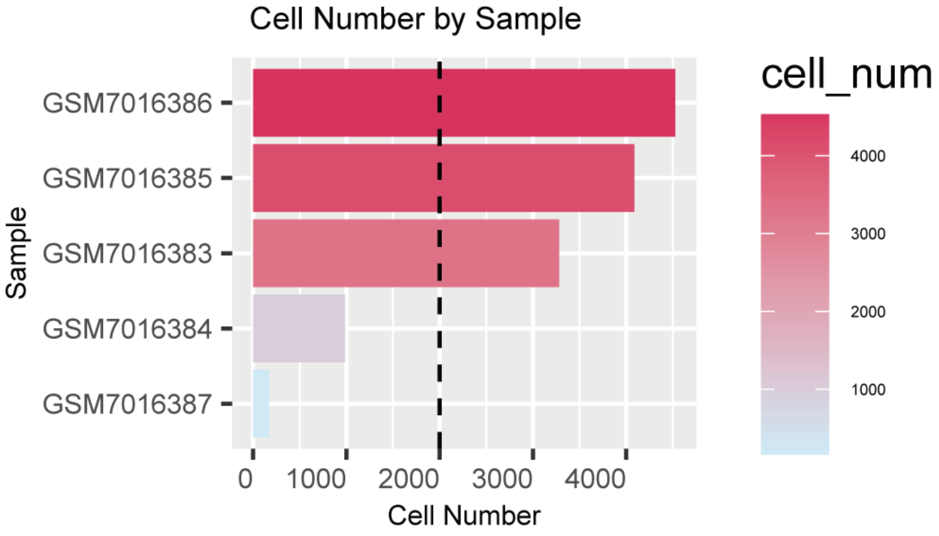

Supplementary Figure2. Cell number quality control of 5 samples in the GSE224143 dataset.

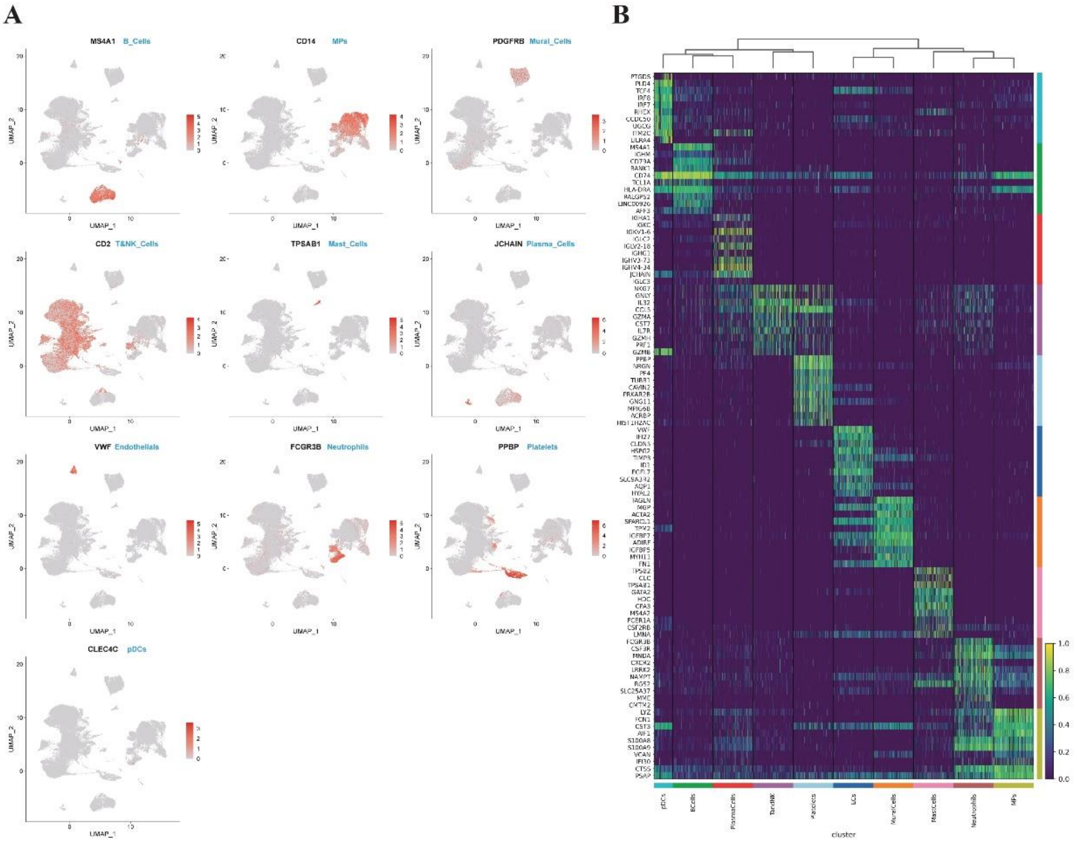

Supplementary Figure3. (A) Projection of representative marker genes and their unified expression levels for each cell type on the UMAP plot. **(B)** Heatmap of the top 10 differentially expressed genes for each cell type.

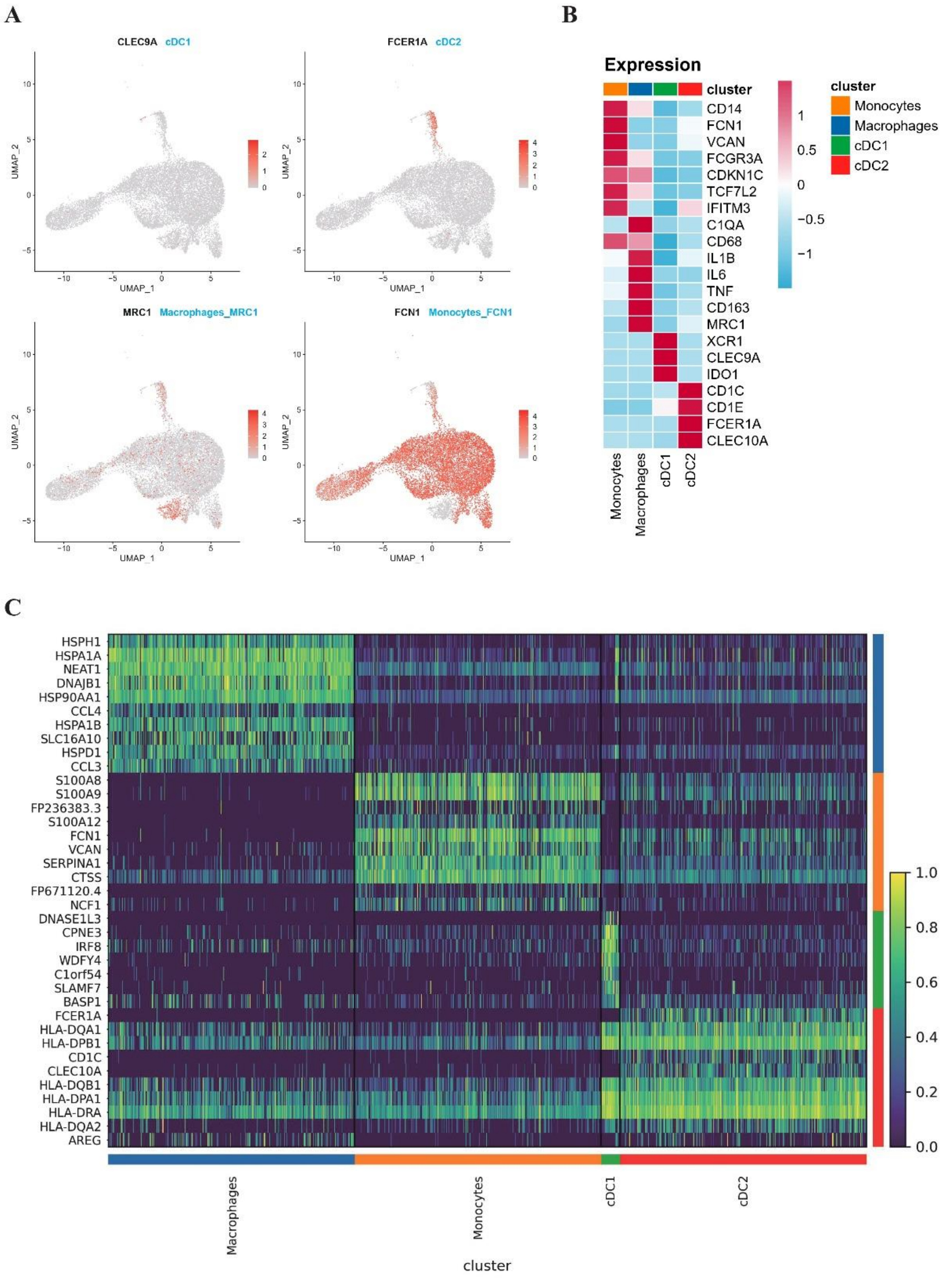

Supplementary Figure4. (A) Projection of representative marker genes and their unified expression levels for macrophages, monocytes, cDC1, and cDC2 cell types on the UMAP plot. **(B)** A heatmap was used to annotate marker genes for macrophages, monocytes, cDC1 and cDC2. **(C)** A heatmap displaying the top 10 differentially expressed genes between the four cell types.

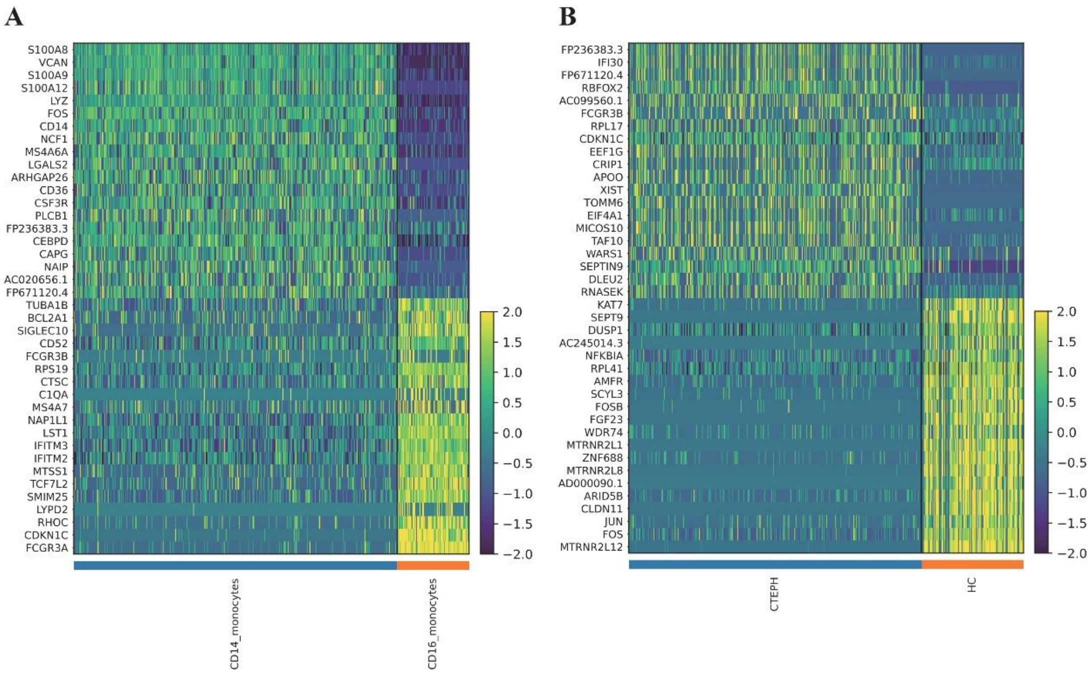

Supplementary Figure5. (A) Heatmap of the top 20 differentially expressed genes between CD14+ monocytes and CD16+ monocytes in CTEPH patients. **(B)** Heatmap of the top 20 differentially expressed genes in CD16+ monocytes between CTEPH patients and the HC group.

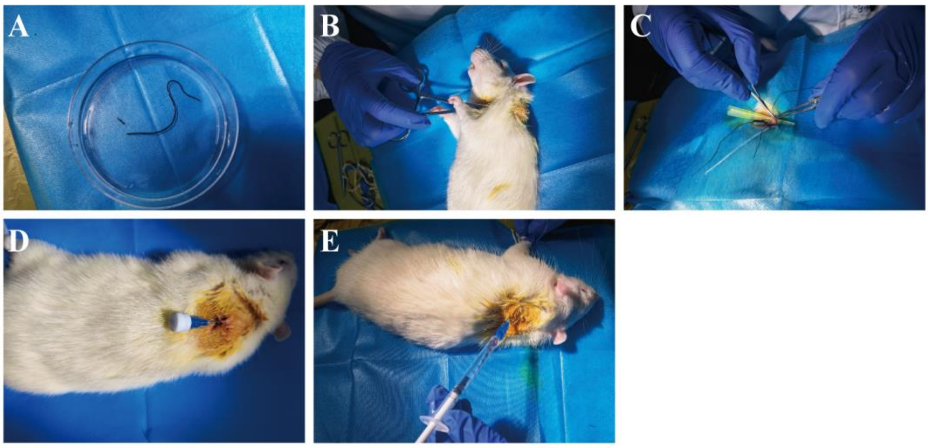

Supplementary Figure6. Brief description of the surgical procedure for CTEPH rat construction

(A) Prepare rat autologous thrombus; **(B)** Construct a subcutaneous tunnel from the rat's neck to the back; **(C)** Isolate the rat's left external jugular vein, ligate the distal end with a suture, and clamp the proximal end with a vascular clamp. The central venous catheter is inserted through the external jugular vein; **(D)** Fix the central venous catheter on the rat's back, suture the back incision and disinfect; **(E)** Inject rat autologous thrombus through the central venous catheter.

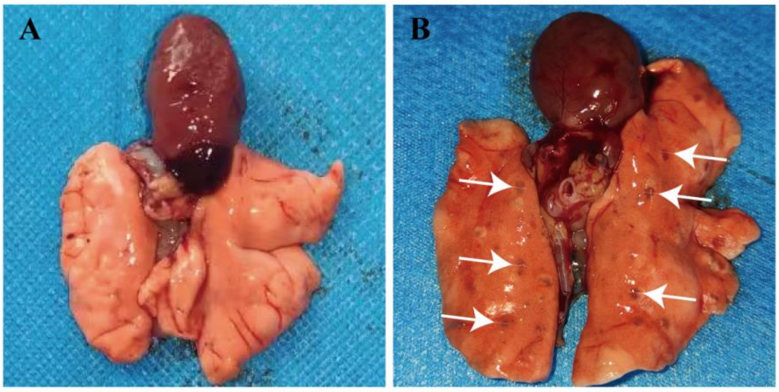

Supplementary Figure7. Lung and heart tissues of rats in the sham surgery group and CTEPH group.

(A) The lung tissue of rats in the sham surgery group was smooth on the surface, and the heart tissue was conical. **(B)** Scattered reddish-brown spots (white arrows) were visible on the lung tissue of CTEPH rats, and the heart tissue was pear-shaped.

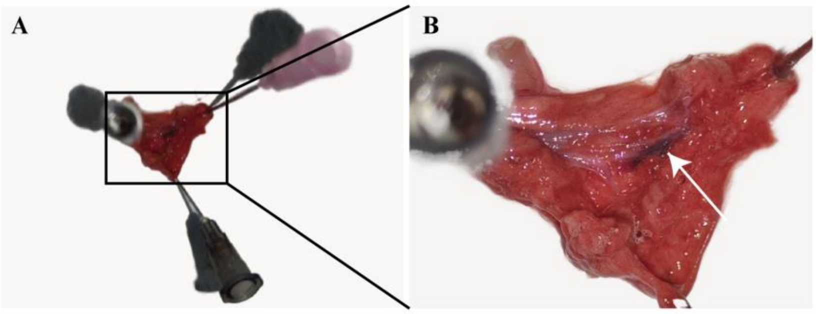

Supplementary Figure8. Thrombus (white arrow) was visible in the pulmonary artery of CTEPH rats.

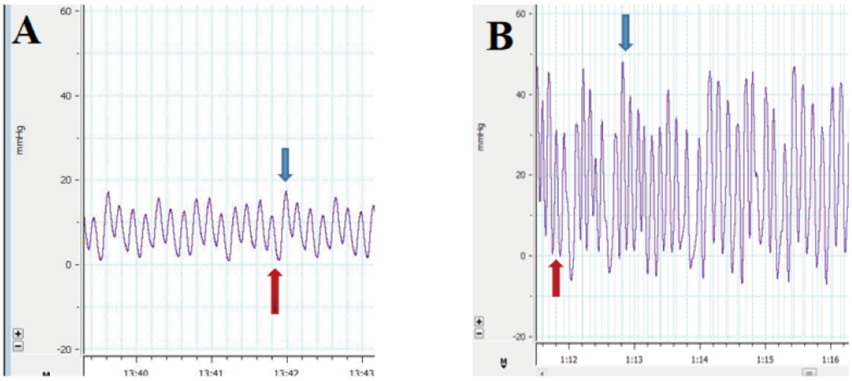

Supplementary Figure9. The graph shows the right ventricular pressure (RVP) curves of rats.

(A) Control group. **(B)**CTEPH group. The blue arrows indicate the right ventricular systolic pressure, and the red arrows indicate the right ventricular diastolic pressure. (RVP: Right Ventricular Pressure).

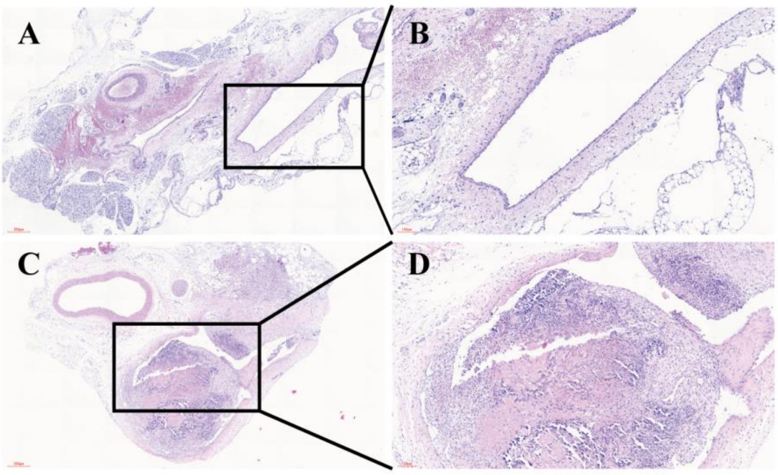

Supplementary Figure 10. H&E staining of rat inferior vena cava.
(A-B) H&E staining of inferior vena cava and surrounding tissue vessels of rats in the Sham group. Scale bar in A: 300μm; Scale bar in B: 100μm. **(C-D)** Inferior vena cava and surrounding tissue vessels of rats in the DVT group, with thrombus formation in the inferior vena cava. Scale bar in C: 300μm; Scale bar in D: 100μm.

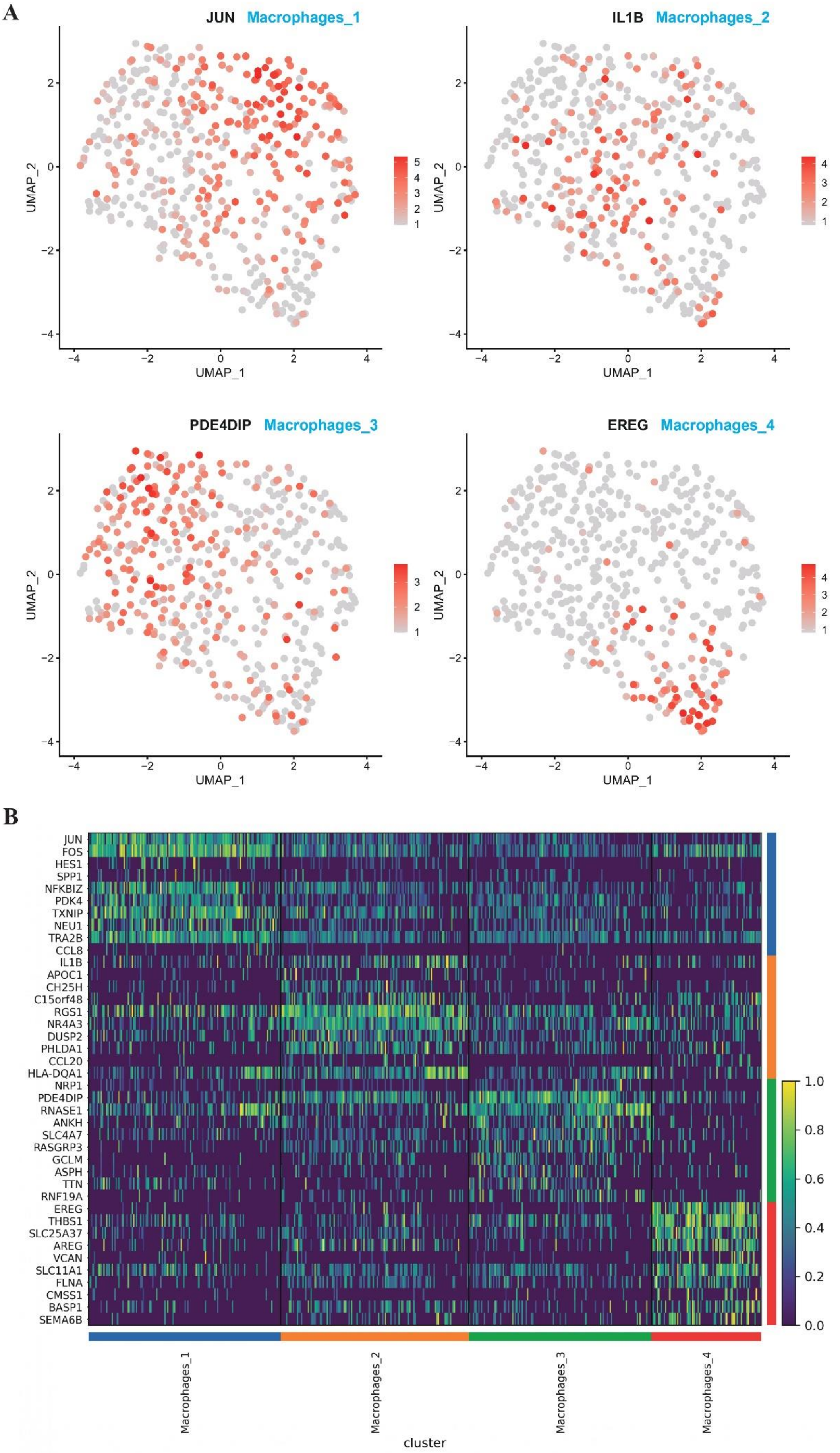
A
JUN Macrophages_1
IL1B Macrophages_2
PDE4DIP Macrophages_3
EREG Macrophages_4
UMAP_1
UMAP_2
B
JUN
FOS
HES1
SPP1
NFKBIZ
PDK4
TXNIP
NEU1
TRA2B
CCL8
IL1B
APOC1
CH25H
C15orf48
RGS1
NR4A3
DUSP2
PHLDA1
CCL20
HLA-DQA1
NRP1
PDE4DIP
RNASE1
ANKH
SLC4A7
RASGRP3
GCLM
ASPH
TTN
RNF19A
EREG
THBS1
SLC25A37
AREG
VCAN
SLC11A1
FLNA
CMSS1
BASP1
SEMA6B
1.0
0.8
0.6
0.4
0.2
0.0
Macrophages_1
Macrophages_2
Macrophages_3
Macrophages_4
cluster

Supplementary Figure11. (A) Projection of typical marker genes and their unified expression levels for the four macrophage subgroups on the UMAP plot. **(B)** Heatmap of the top 10 differentially expressed genes in each macrophage subset.

A

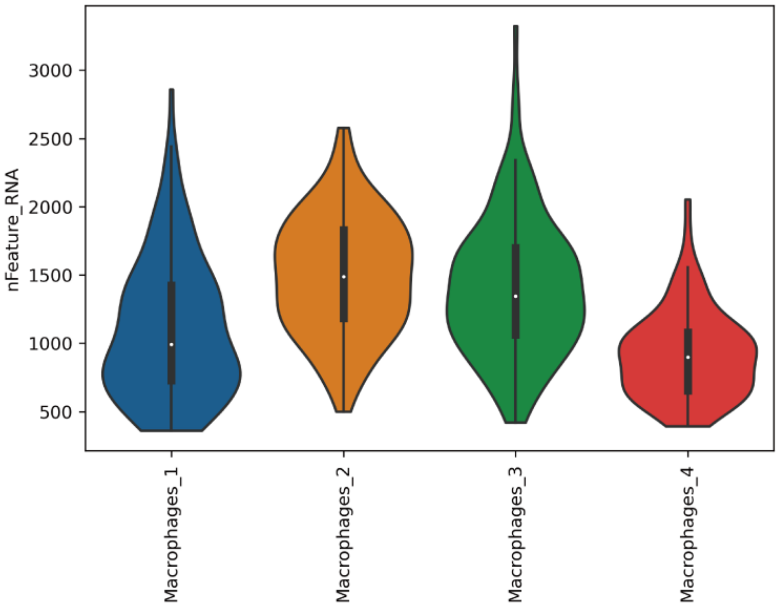

B

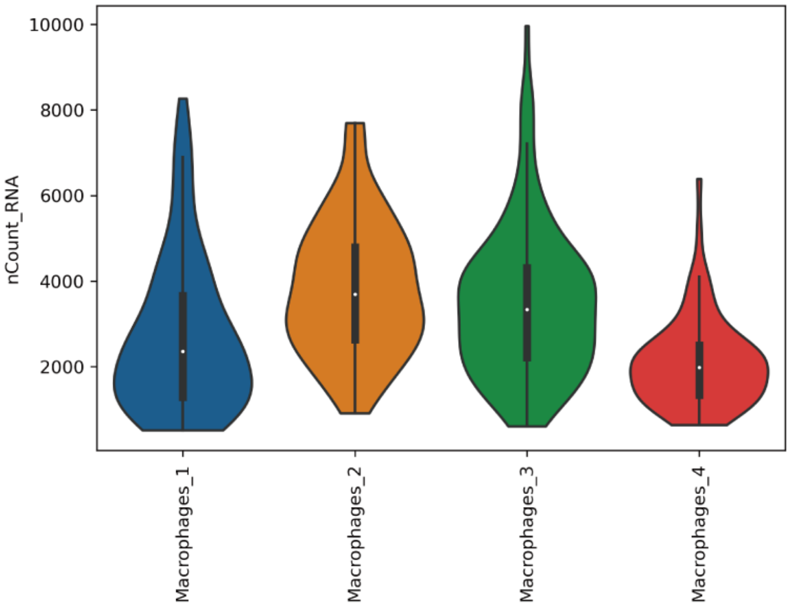

Supplementary Figure12. (A) Transcript expression abundance of each macrophage subset. **(B)** The number of genes expressed in each macrophage subset.

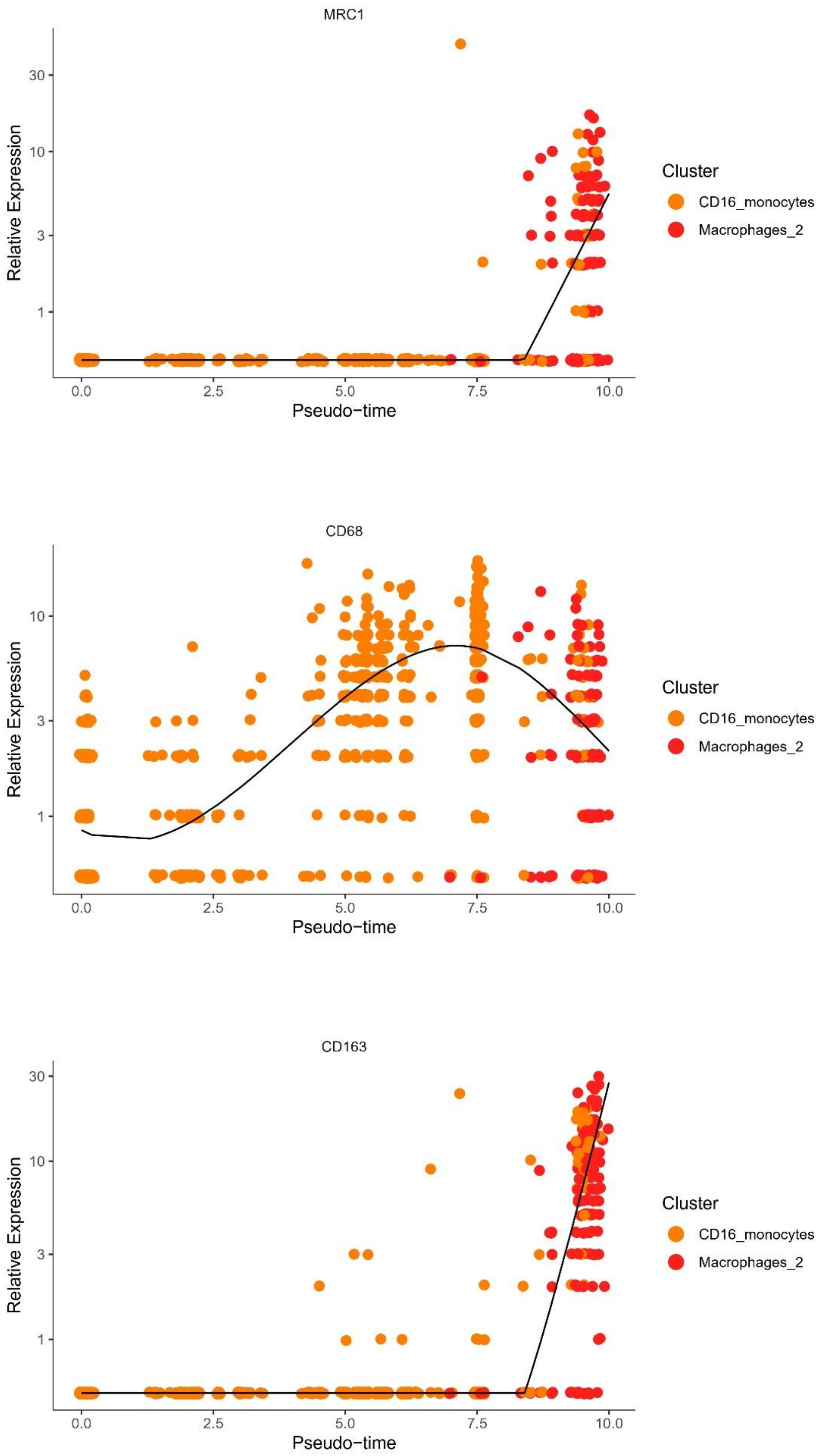

Supplementary Figure13. Expression trends of macrophage marker genes CD68, CD163, and MRC1 along the developmental trajectory.

Supplementary Table1. Clinical characteristics of five CTEPH patients who provided peripheral blood for scRNA-seq in this study.

	CTEPH-1	CTEPH-2	CTEPH-3	CTEPH-4	CTEPH-5
Group	CTEPH-N	CTEPH-I	CTEPH-I	CTEPH-N	CTEPH-N
Gender	Female	Male	Female	Female	Female
Age(year)	54	50	50	59	60
BMI(kg/m^2)	21.5	23.7	25	23.3	21.5
NYHA class	III	I	I	II	II
6MWD(m)	326	486	480	418	450
Disease duration(year)	1	2	2	4	2
History of acute PE/DVT	Yes	Yes	Yes	Yes	Yes
RA(mmHg)	6	3	4	5	4
Mean PA pressures(mmHg)	54	28	26	64	48
PVR(WU)	16	2.5	3.1	11.0	9.3
Cardiac Index($L*min^{-1}*m^{-2}$)	1.4	3.3	3.0	2.7	1.8
pulmonary endarterectomy	No	Yes	No	No	No
Balloon Pulmonary Angioplasty	No	No	Yes	No	No
PDE5 inhibitors	Sildenafil	-	-	-	-
sGC Inhibitor	-	Riociguat	Riociguat	-	Riociguat
Prostacyclin analogs	Selexipag	-	-	-	-
Endothelin receptor antagonists	Macitentan	Macitentan	Bosentan	Ambrisentan	Ambrisentan

Supplementary Table2. Baseline data and clinical features of 15 CTEPD patients and paired healthy controls who provided peripheral blood for flow cytometry analysis in this study.

	CTEPD (n=15)	HC (n=15)
Gender, female	12	10
Age(year)	59.73±10.71	57.07±9.92
BMI(kg/m^2)	24.43±8.23	23.13±3.26
Smoking	1	0
Family history of venous thromboembolism(%)	0	0
Hypertension	5	-
WHO functional class Ⅰ-Ⅱ	7	-
WHO functional class Ⅲ-Ⅳ	8	-
Disease duration(year)	3.6±3.47	-
History of acute PE/DVT	14	-
Use of PH targeting drugs	11	-

Supplementary Table3. The markers of resident macrophage genes

Gene name
MRC1
CD163
CD163L1
FOLR2
HES1
